# YX-112, a novel celastrol-derived PROTAC, inhibits the development of triple-negative breast cancer by targeting the degradation of multiple proteins

**DOI:** 10.3389/fphar.2025.1571135

**Published:** 2025-04-15

**Authors:** Yongxue Gu, Mengmeng Yang, Wenbin Wang, Lihua Li, Ying Ma, Wenshan Liu, Qiang Zhao

**Affiliations:** ^1^ Thyroid and Breast Medical Center, Weifang People’s Hospital, Shandong Second Medical University, Weifang, Shandong, China; ^2^ Clinical Research Center, Affiliated Hospital of Shandong Second Medical University, Weifang, Shandong, China; ^3^ School of Pharmacy, Tianjin Medical University, Tianjin, China; ^4^ Shandong Key Laboratory of Medicine and Health (Clinical Applied Pharmacology), Department of Pharmacy, Affiliated Hospital of Shandong Second Medical University, Weifang, Shandong, China; ^5^ Department of Anesthesiology, Weifang People’s Hospital, Shandong Second Medical University, Weifang, Shandong, China

**Keywords:** celastrol, protac, DIA-based quantitative proteomics, triple-negative breast cancer, molecular docking

## Abstract

**Background:**

Celastrol is an effective component of the plant Tripterygium wilfordii Hook. f., which has a high inhibitory effect on triple-negative breast cancer. However, the therapeutic window of celastrol is narrow, and as a multi-target drug, its mechanism of action in triple-negative breast cancer is not very clear. Therefore, developing new celastrol derivatives has become an urgent task.

**Method:**

In this work, we apply the PROTAC strategy to design and synthesis novel celastrol derivative. The antiproliferative activity of compound YX-112 against various types of cells was detected by CCK8 method. DIA-based quantitative proteomics, Western blot was used to explore the mechanism of compound YX-112 on triple-negative breast cancer MDA-MB-231 cells. Finally, the binding mode between compound YX-112 and target protein was predicted through molecular docking.

**Results:**

We developed a novel PROTAC YX-112 of celastrol, which was extremely sensitive to the triple-negative breast cancer MDA-MB-231 cells, with an IC_50_ value of 0.32 ± 0.04 μM, and its antiproliferative activity was 3 times that of celastrol. Subsequently, through DIA-based quantitative proteomics and Western blot validation experiments, it was found that YX-112 could target the degradation of CHEK1 and PIK3R2 proteins in MDA-MB-231 cells in a ubiquitin-proteasome dependent manner, indicating that it could be used as a degrader of CHEK1 and PIK3R2 proteins. Additionally, YX-112 could effectively inhibit the expression levels of CDK4 and p-AKT, and its inhibitory effect was stronger than that of celastrol. Finally, molecular docking predicted the binding mode between celastrol and CHEK1, showing that celastrol could form hydrogen bond interaction with the key residue GLN13.

**Conclusion:**

This study provides new insights into the derivation of celastrol and its molecular mechanisms of action.

## 1 Introduction

Cancer is the second deadliest disease globally. According to statistics, there would be nearly 20 million new cancer cases and approximately 9.7 million cancer deaths worldwide in 2022. Among them, Breast cancer (BC) is the second malignant tumor in incidence rate. For women, breast cancer is the most common cancer. In 2022, there would be 2.3 million new breast cancer patients and 670,000 deaths worldwide, which had become the biggest challenge for women’s health (Bray et al., 2024; Giaquinto et al., 2024). Based on molecular markers, including estrogen receptor (ER), progesterone receptor (PR) and human epidermal growth factor receptor 2 (HER2), breast cancer can be divided into three subtypes: hormone receptor (HR) positive, HER2 positive and triple-negative breast cancer (TNBC). Among them, TNBC accounts for about 15%–20% of all breast cancer, which is generally more invasive than other types of breast cancer, more prone to metastasis, and has a poor prognosis (Loibl et al., 2021; Yin et al., 2020). At present, the treatment of triple-negative breast cancer includes surgery, radiotherapy, chemotherapy and targeted therapy. Drug resistance and postoperative metastasis are the main causes of death of breast cancer (Wang and Wu, 2023; Khan et al., 2024). Therefore, it has always been an important research work to find new and effective strhategies to treat triple-negative breast cancer.

Natural drugs are natural products extracted from plants, fungi, or marine organisms that exist in nature. Compared with traditional chemotherapy drugs, natural medicines exhibit unique advantages in anticancer activity, toxic side effects, and overcoming drug resistance (Huang et al., 2021; Baell, 2016). Currently, 80% of the small molecule anticancer drugs approved by the FDA are natural drugs and their derivatives, such as paclitaxel, camptothecin, etc., (Luo et al., 2024). Therefore, using natural products as lead compounds for structural derivation is an important approach to the development of anticancer drugs. Celastrol (CEL) is one of the active ingredients in the plant Tripterygium wilfordii Hook. f., belonging to the suberin type pentacyclic triterpenoids. Celastrol has a variety of pharmacological activities, including anti-tumor, anti-inflammatory, anti-virus and antiobesity, especially for breast cancer, lung cancer, colorectal cancer, etc., (Hou et al., 2020; Fang et al., 2019; Wang C. et al., 2023). Among them, celastrol has attracted attention in the treatment of triple-negative breast cancer.

Celastrol can exert anticancer effects by inhibiting cancer cell proliferation, migration, and invasion, inducing cancer cell apoptosis and autophagy, inhibiting angiogenesis, and suppressing tumor metastasis. It can also regulate multiple cancer-related signaling pathways, including PI3K/Akt/mTOR, JAK-STAT, Wnt/β-catenin, NF-κB, JNK/Nrf2/HO-1 and AMPK (Wang C. et al., 2023; Shi et al., 2020). As a multi-target anticancer drug, the direct targets of celastrol include heat shock protein 90 (HSP90), peroxidized reductase (PRDX) family proteins, histone deacetylase (HDAC), STAT3, etc., (Lu et al., 2010; Xu et al., 2023; Wang S. et al., 2023; Xu et al., 2022). However, the molecular mechanism of celastrol in triple-negative breast cancer is not very clear, so it is necessary to explore its molecular mechanism. Proteolysis targeting chimera (PROTAC) is currently one of the most popular drug development technologies, which can selectively degrade target proteins through the ubiquitin proteasome system. As a bifunctional molecular compound, PROTAC consists of three parts, including a ligand that binds to the target protein, a ligand that binds to ubiquitin ligase E3, and a linker that connects the two (Li and Song, 2020). PROTAC molecules have significant prospects in enhancing drug activity, reducing toxicity, and improving selectivity. It is noteworthy that the combined application of PROTAC technology and proteomics technology can be used to identify the potential targets of active natural products, which helps to reveal the potential targets of celastrol in triple-negative breast cancer.

**FIGURE 1:**
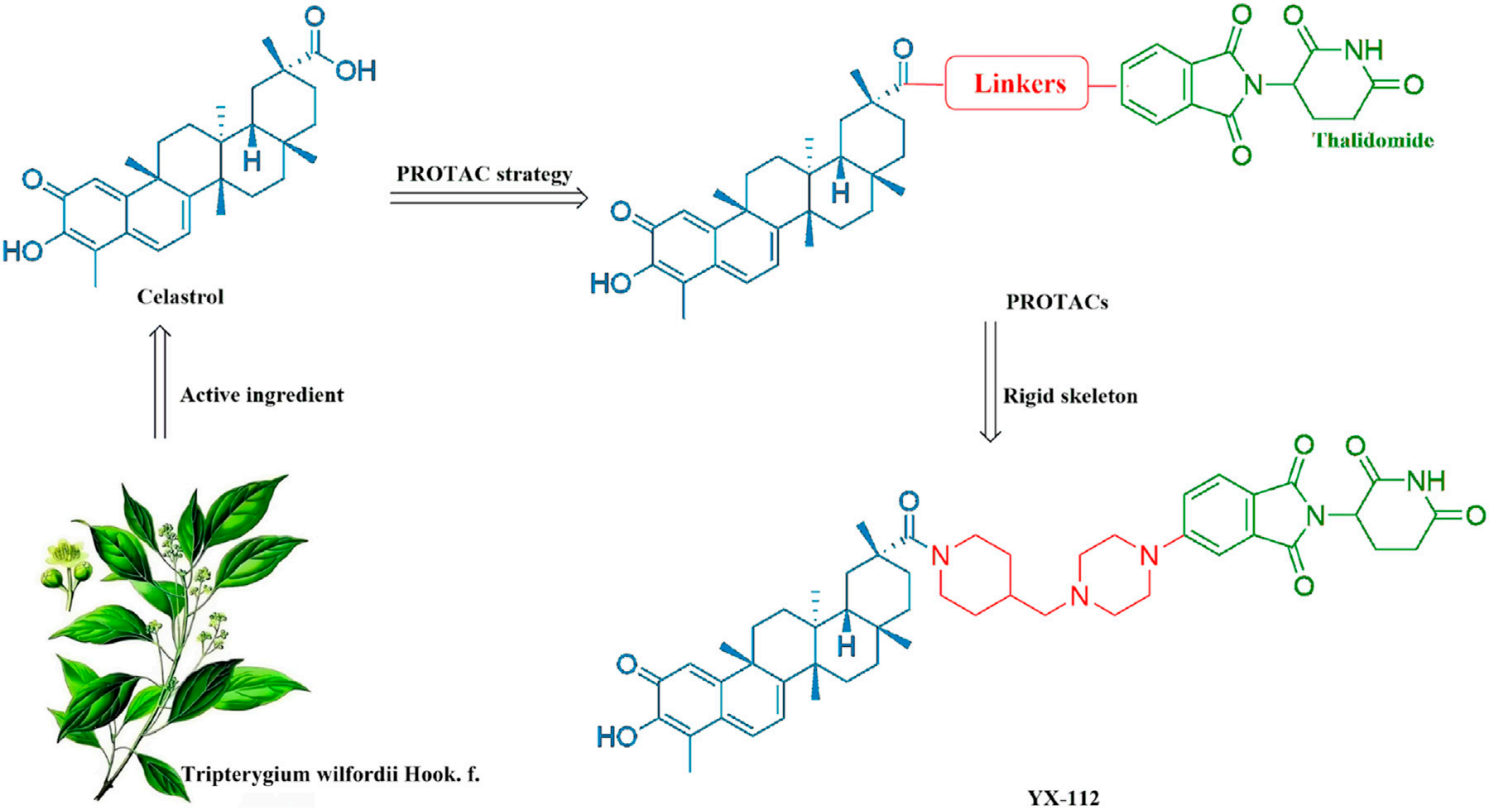
Design strategy of the celastrol-based PROTAC YX-112.

Previously, researchers have reported partial PROTACs of celastrol, but the linkers they chose were all flexible skeletons, lacking rigid skeletons, which often resulted in poor water solubility (Ni et al., 2024; Gan et al., 2024). In this study, we applied PROTAC technology to design novel PROTAC molecules of celastrol. Compound YX-112 was designed and synthesized by using thalidomide as the E3 ligase ligand and innovatively introducing a rigid skeleton connected by pyridine and piperazine rings as the linker (Figure 1). The cell activity test showed that compound YX-112 was highly sensitive to triple-negative breast cancer MDA-MB-231 cells and showed strong antiproliferative activity. Subsequently, quantitative proteomic analysis based on data-independent acquisition (DIA) was performed on MDA-MB-231 cells treated with YX-112 to further explore the molecular mechanism of YX-112 in triple-negative breast cancer and reveal its target. This study provides a new idea for the derivation of celastrol and its mechanism of action against triple-negative breast cancer.

## 2 Materials and methods

### 2.1 Chemistry

Celastrol (>98%) was purchased from Shanghai Bidde Medical Technology Co., Ltd. (China). The preparation method and structural confirmation of compound YX-112 were showed in Supplementary Scheme S1.

### 2.2 CCK8 experiment

MDA-MB-231 cells, 4T1 cells, NCI-H1975 cells, SW620 cells and HK-2 cells were obtained from Procell Life Science & Technology Co., Ltd. (Wuhan, China). Cells were seeded in 96-well plates at 5,000 cells/well. After the cells were in good condition, different concentrations of compounds (0.016, 0.08, 0.4, 2, 10, and 50 μM) were added and incubated in the cell incubator for 72 h. Each well contained 100 μL of medium and 10 μL of CCK8 solution (Beyotime Biotechnology, Shanghai, China) was added according to the instructions. The incubation was continued in the cell incubator for about 1 h, and the absorbance was measured at a wavelength of 450 nm using a microplate reader.

### 2.3 DIA-based quantitative proteomics

MDA-MB-231 cells were seeded with a density of 2 million cells per dish in a 100 mm culture dish, with a total of 6 dishes of cells, and incubated overnight. Then, the target compound and blank control DMSO were added separately and stimulated for 24 h, and three biological replicates were set in each group. Then the cells were collected and washed three times with PBS. Then, DIA-based proteomics analysis was conducted using the proteomics platform of Beijing Biotech-Pack Scientific Co., Ltd.

### 2.4 Western blot experiment

MDA-MB-231 cells were seeded in 6-well plates at 5 × 10^5^ cells/well. After the cells were in good condition, different concentrations (0, 0.1, 0.2, and 0.4 μM) of the compounds were added to stimulate the cells for 24 h, and the concentration of the positive control was 0.4 μM. The cells were collected into centrifuge tubes by adding RIPA buffer using a cell scraper and lysed for 30 min on ice. The extracted protein was quantified by BCA protein quantification kit (Beyotime Biotechnology, Shanghai, China), and then Loading Buffer was added and boiled at 100°C for 10 min. Proteins were separated in 10% SDS-PAGE and electrotransferred to PVDF membranes. The PVDF membranes were blocked with 5% skimmed milk (Tris-buffered saline containing 0.05% Tween20) for 2 h. The bands were incubated with primary antibodies CDK4 (1:800, 11,026, Proteintech), CHEK1 (1:800, 25,887, Proteintech), PIK3R2 (1:800, 67,644, Proteintech), Akt (1:800, 9,272, CST), Phospho-Akt (1:800, 4,060, CST), and β-actin (1:2,000, AB0035, Abways) for more than 8 h at 4°C. The secondary antibody was then incubated at room temperature for 1 h. Finally, the blots were detected using the Omni-ECL™ ultra-sensitive chemiluminescence detection kit (Epizyme Biotech, Shanghai, China).

### 2.5 Molecular docking

The molecular docking process between CHEK1 and celastrol was completed through discovery studio v3.5 software. Firstly, the crystal structure of CHEK1 (ID: 4FSN) was downloaded from the protein data bank. The crystal structure was prepared through the “protein preparation” module in the software, including removing water, adding hydrogen atoms, adding missing residues and so on. The docking pocket was constructed using ligands inherent in the crystal structure. Then, the structure of celastrol was imported into DS software using Chem 3D software, and its preparation work was completed, including maintaining ionization, desalination, and forming tautomers. Finally, the docking of celastrol with CHEK1 was completed using the -CDOCKER program, and the binding information between them was analyzed based on the docking result.

## 3 Results and discussion

### 3.1 Cell viability evaluation

**FIGURE 2:**
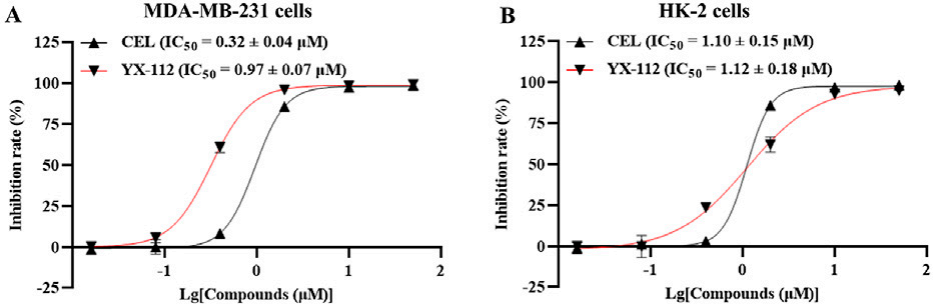
Cell viability detection of compound YX-112 and celastrol. **(A)** MDA-MB-231 cells were treated with compound 112 and celastrol for 3 days, respectively, and the inhibitory activity was detected; **(B)** HK-2 cells were treated with compound 112 and celastrol for 3 days, respectively, and the inhibitory activity was detected. All data were presented as mean ± SD for three statistical experiments.

The antiproliferative activity of compound YX-112 and celastrol on different cancer cells were tested by CCK8 method, including triple-negative breast cancer MDA-MB-231 cells and 4T1 cells, human non-small cell lung cancer NCI-H1975 cell, and human colorectal cancer SW620 cells. Table 1 displayed that compared with celastrol, compound YX-112 had a stronger antiproliferative effect on these cancer cells, demonstrating a broad-spectrum anticancer effect. It was worth noting that compound YX-112 was more sensitive to triple-negative breast cancer cells, and has stronger antiproliferative activity against MDA-MB-231 cells and 4T1 cells. Among them, compound YX-112 had IC_50_ values of 0.32 ± 0.04 μM on MDA-MB-231 cells, and its inhibitory activity was 3 times that of celastrol (Figure 2A). In addition, we also tested the inhibitory activity of compounds YX-112 and celastrol on normal human renal epithelial cells HK-2 and evaluated their cytotoxicity. The IC_50_ value of compound YX-112 on HK-2 cells was 1.12 ± 0.18 μM, and its cytotoxicity was similar to that of celastrol (Figure 2B). Their selectivity index (SI) for HK-2 and MDA-MB-231 cells were calculated. The SI of YX-112 was found to be 3.50, which was significantly higher than that of celastrol, indicating that it had a certain targeting selectivity for triple-negative breast cancer MDA-MB-231 cells. Based on this, compound YX-112 had potential value in the treatment of triple-negative breast cancer and was worth further exploring its mechanism of action.

**TABLE 1:** Cell viability evaluation of compound YX-112 and celastrol.

Compd	IC_50_ (μM)^a^					SI^b^
	MDA-MB-231	4T1	NCI-H1975	SW620	HK-2	
YX-112	0.32 ± 0.04	0.35 + 0.04	0.37 + 0.04	0.51 + 0.06	1.12 ± 0.18	3.50
Celastrol	0.97 ± 0.07	0.92 ± 0.08	0.75 + 0.08	0.98 + 0.08	1.10 ± 0.15	1.13

^a^ Average of three statistical experiments.

^b^ SI (Selectivity index) = IC_50_ value of HK-2, cells/IC_50_ value of MDA-MB-231, cells.

### 3.2 Identification of the differentially expressed proteins

In order to identify the target of YX-112 in triple-negative breast cancer cells MDA-MB-231 and reveal the molecular mechanism of its action. The DIA-based quantitative proteomic analysis was applied to perform proteomic analysis on MDA-MB-231 cells treated with compound YX-112, and compared with control group (DMSO) cells. A total of 6,744 proteins were identified in this analysis. In the screening process of differentially expressed proteins, a fold change (FC) > 1.5 (or <0.67) was used as the screening criterion. Proteins with FC > 1.5 represented significant upregulation, while proteins with FC < 0.67 represented significant downregulation. Supplementary Table S1 showed the number of differentially expressed proteins. It could be seen that compound YX-112 caused significant changes in the number of proteins, which was 112, including 101 significantly downregulated proteins and 11 significantly upregulated proteins. Supplementary Table S1 listed the protein information of all significant differences detected.

**FIGURE 3:**
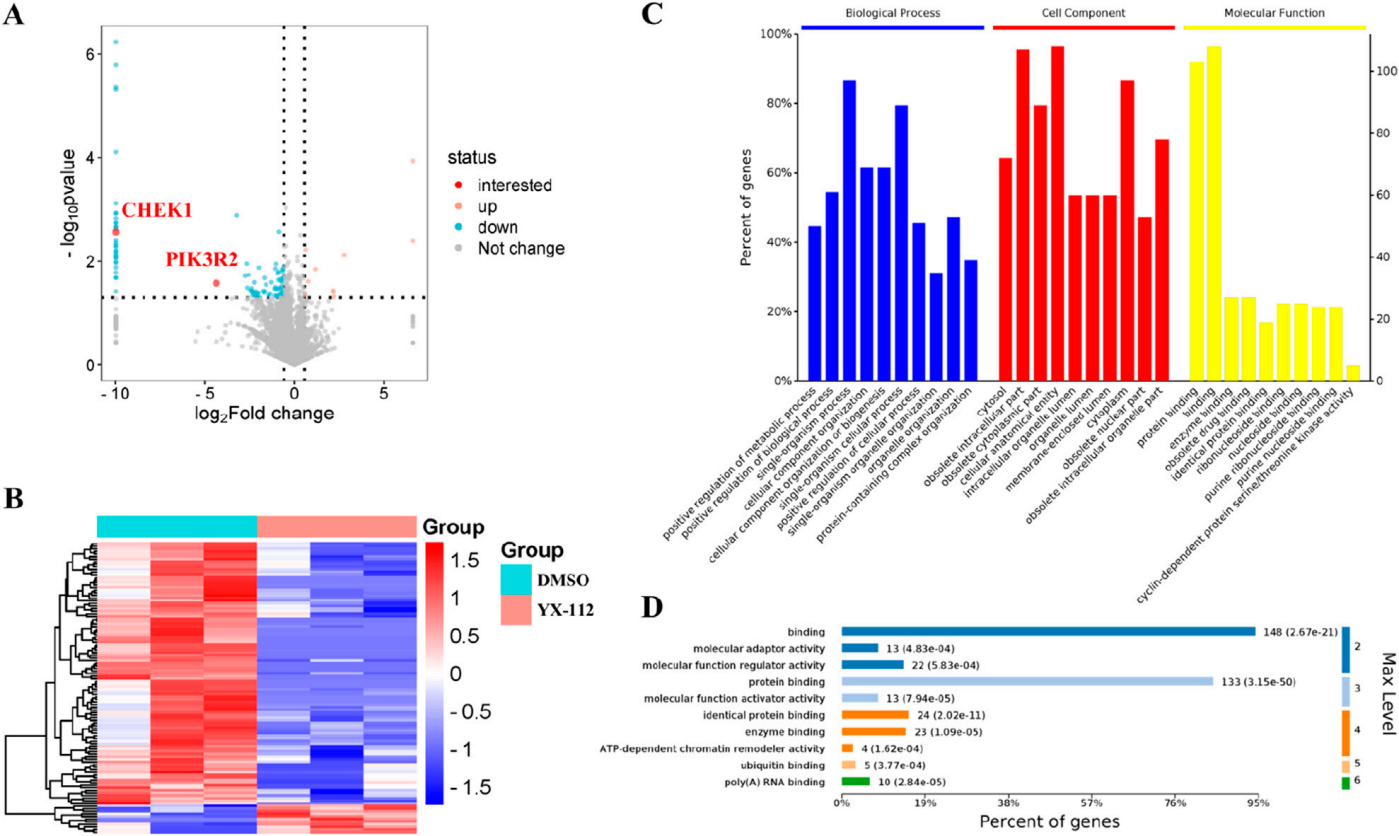
Bioinformatics analysis of differentially expressed proteins between the YX-112 group (0.4 μM) and the control group (DMSO). **(A)** Showed the volcano plot of differentially expressed proteins, in which two significantly downregulated important proteins (CHEK1 and PIK3R2) were highlighted in red; **(B)** showed the cluster heatmap of differentially expressed proteins; **(C)** showed the GO analysis of differentially expressed proteins; **(D)** showed the GO entries significantly enriched at different levels under molecular function.

To compare the significant differences in protein expression between groups, a volcano plot was plotted using fold change and p-value as criteria for comparing the proteins in each group (Figure 3A). The significantly downregulated proteins are shown in blue and red (FC < 0.67 and p < 0.05), the significantly upregulated proteins are shown in orange color (FC > 1.5 and p < 0.05), and the proteins with no difference are shown in gray. The expression proteins between samples were compared using hierarchical cluster algorithm to verify the reliability of biological analysis. The higher the similarity in protein expression within a group, the lower the similarity in protein expression between groups, indicating a more significant difference in protein expression. Cluster heatmap 3B showed the significantly differentially expressed proteins obtained based on the screening criteria of FC > 1.5 times and P value <0.05.

To evaluate the functional significance of these 112 differentially expressed proteins, Gene ontology (GO) enrichment analysis was performed, including biological process (BP), cellular component (CC), and molecular function (MF). The result showed that binding and protein binding are the main molecular functions, single organism process and single organism cellular process are the main biological processes, and cellular anatomical entity and isolated intracellular part are the main cellular components (Figure 3C). In addition, we focused on enriched molecular functions and presented detailed functional information. It was found that there were 108 genes involved in binding and 103 genes involved in protein binding, which played important roles in molecular function (Figure 3D).

### 3.3 KEGG pathway analysis of the differentially expressed proteins

**FIGURE 4:**
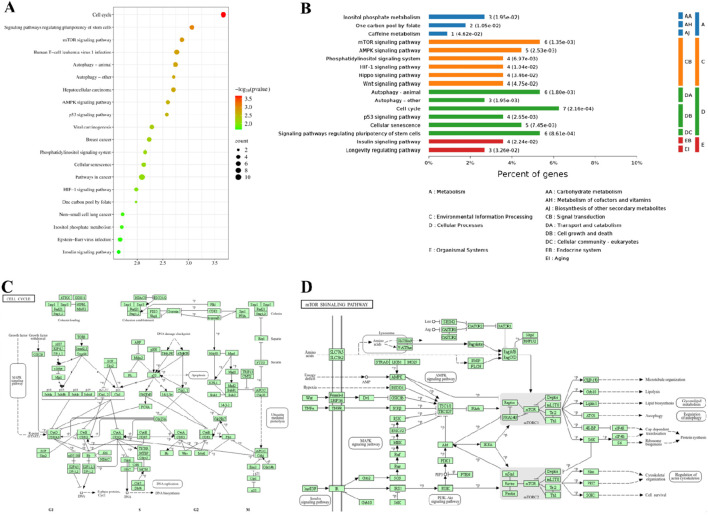
KEGG enrichment analysis of differentially expressed proteins. **(A)** The top 20 KEGG enriched pathways; **(B)** Detailed classification of KEGG enriched pathways; **(C)** KEGG enrichment pathway diagram of cell cycle process; **(D)** KEGG enrichment pathway diagram of mTOR signaling pathway.

To investigate the mechanism of action of compound YX-112 on MDA-MB-231 cells, we conducted an enrichment analysis of differentially expressed proteins based on the KEGG pathway. By conducting KEGG pathway enrichment analysis on differentially expressed proteins, the top 20 significant pathways were identified, including cell cycle, mTOR signaling pathway, p53 signaling pathway, and other cancer-related pathways (Figure 4A). Subsequently, we performed specific biological classification on the enriched pathways (Figure 4B). It was known that the number of proteins enriched in the cell cycle was 7, and the number of proteins enriched in the mTOR signaling pathway was 6, indicating that compound YX-112 mainly affected these two cancer pathways. Subsequently, we demonstrated the KEGG enrichment pathways of these two key pathways, which helped to gain a deeper understanding of their mechanisms of action (Figures 4C,D). The clear version of cell cycle and mTOR signaling pathway could also be found separately on the KEGG pathway database (https://www.kegg.jp/pathway/hsa04110 and https://www.kegg.jp/pathway/hsa04150).

### 3.4 Protein-protein interaction network analysis of the differentially expressed proteins

**FIGURE 5:**
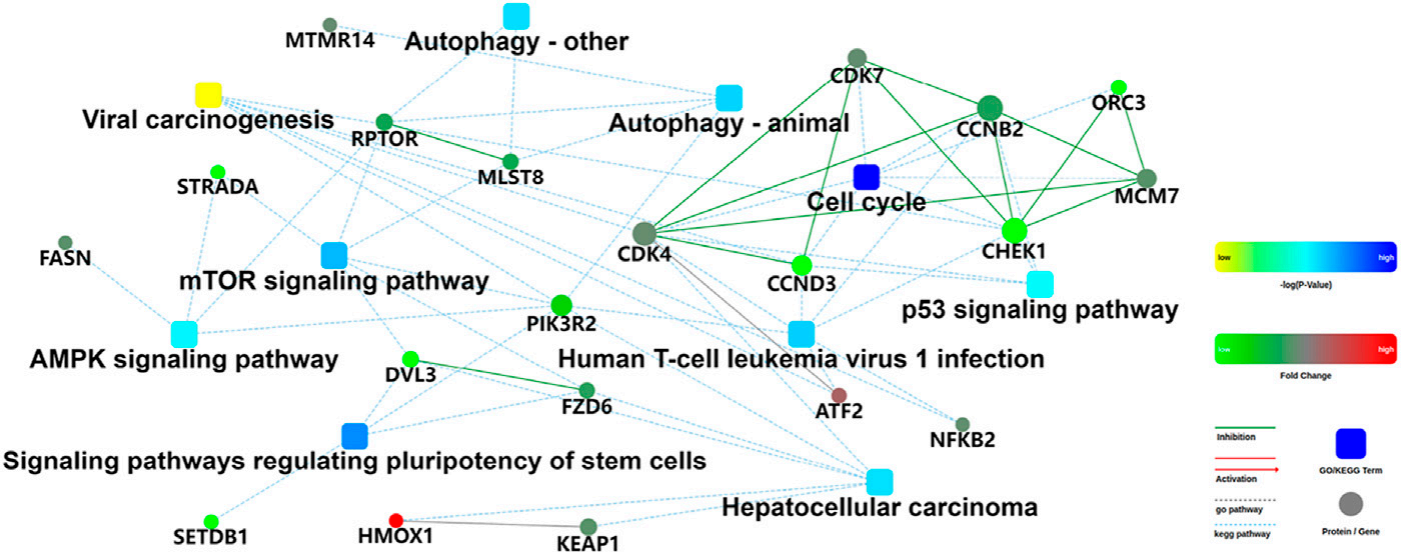
Protein-protein interaction network of the differentially expressed proteins. The box represented GO/KEGG enrichment entries, and the color represented statistical probability; The dots represented proteins/genes, and the color represented fold change; The green line represented inhibition, the red line represented activition, the gray dashed line represented GO database enrichment, and the blue dashed line represented KEGG database enrichment.

One of the important ways in which proteins function was by interacting with other proteins and exerting biological regulatory effects through protein-protein mediated pathways. Proteins with high connectivity might be key points in signal transduction pathways. Therefore, protein-protein interaction (PPI) network analysis had important research value. In addition, the combination of the protein-protein interaction network and KEGG pathway could obtain more detailed molecular mechanisms (Athanasios et al., 2017; Wang et al., 2022). Subsequently, the differentially expressed proteins were imported into the STRING database, and a protein-protein interaction network analysis graph was obtained (Figure 5). It was evident that the cell cycle and mTOR signaling pathways played important roles. Among them, checkpoint kinase 1 (CHEK1) was a key protein in the cell cycle and p53 signaling pathway, with the highest node degree, and was the most important hub protein among all differentially expressed proteins. Proteins related to the cell cycle also included cyclin-dependent kinase 4 (CDK4), cyclin-dependent kinase 7 (CDK7), minichromosome maintenance protein 7 (MCM7), cyclin D3 (CCND3), cyclin B2 (CCNB2), and Origin recognition complex subunit 3 (ORC3). Additionally, phosphatidylinositol 3-kinase regulatory subunit beta (PIK3R2) was a key protein in the mTOR signaling pathway and also had a high node degree. Proteins associated with the mTOR signaling pathway included Regulatory-associated protein of mTOR (RPTOR), mammalian lethal with SEC13 protein 8 (MLST8), dishevelled-3 (DVL3), frizzled homolog 6 (FZD6), and STE20-related kinase adaptor alpha (STRADA). In summary, the protein-protein interaction network demonstrated in detail the potential association between target proteins and signaling pathways.

### 3.5 Validation of target and signaling pathways

**FIGURE 6:**
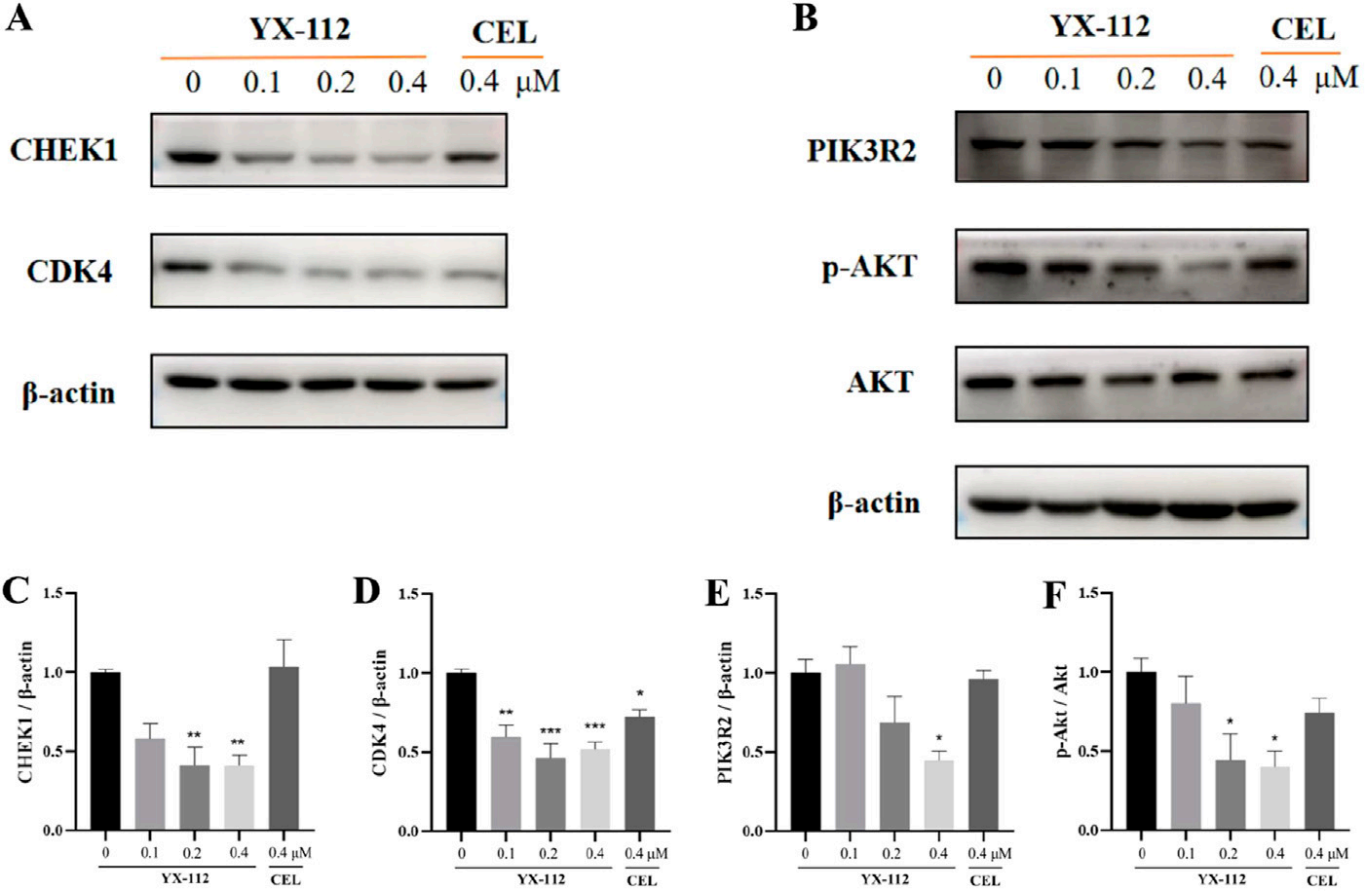
Validation of the mechanism of action of compound YX-112 on MDA-MB-231 cells. **(A, B)** MDA-MB-231 cells were treated with YX-112 (0, 0.1, 0.2, and 0.4 μM) and celastrol (0.4 μM), and the protein expression of CHEK1, CDK4, PIK3R2, p-AKT and AKT was detected; **(C–F)**. Quantitative analysis of three independent experiments. Compared with DMSO group, *P < 0.05, **P < 0.01, ***P < 0.001.

Based on proteomic analysis, we found that CHEK1 and PIK3R2 were proteins significantly associated with cell cycle and mTOR pathway. Here, CHEK1 played a crucial role in the DNA damage response pathway, making it an attractive target for cancer treatment (Hu et al., 2024). PIK3R2 was one of the family members of phosphatidylinositol 3-kinase (PI3K). The high expression of PI3K kinase was usually associated with various cancers, mainly affecting the PI3K-AKT-mTOR pathway. Currently, multiple PI3K inhibitors had been successfully launched on the market (Liu et al., 2022; Peng et al., 2024). Therefore, we further applied Western blot technology to verify whether compound YX-112 could induce degradation of these proteins. The results showed that compound YX-112 could induce the degradation of CHEK1 and PIK3R2, while there was no change in the celastrol group (Figures 6A–C, E). In addition, proteasome inhibitor MG132 could block the degradation of CHEK1 and PIK3R2, indicating that compound YX-112 was dependent on the ubiquitin-proteasome system (Supplementary Figure S1). Therefore, CHEK1 and PIK3R2 were identified as degradation target proteins of YX-112, indicating that they are potential binding proteins for celastrol. Among them, CHEK1 protein could be significantly degraded after treatment with compound YX-112, with a degradation rate of nearly 60% at a concentration of 0.2 μM.

We also detected the expression of CDK4 related to the cell cycle and found that YX-112 and celastrol could induce downregulation of CDK4 (Figures 6A,D). In addition, the PI3K-AKT-mTOR was a classic cancer pathway, and detection has found that compound YX-112 could downregulate the phosphorylation level of AKT, indicating that it could affect the anticancer effect of this pathway (Figures 6B,F). Obviously, YX-112 had a significantly stronger inhibitory effect on CDK4 and p-AKT than celastrol, which was consistent with cell proliferation activity.

### 3.6 Prediction of docking mode

**FIGURE 7:**
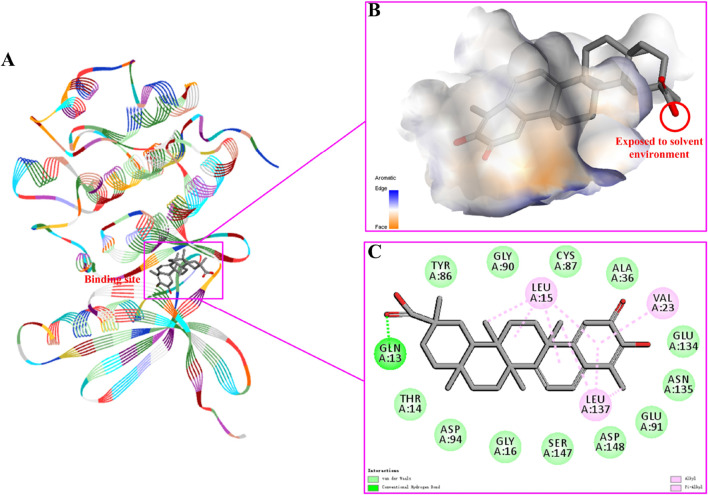
Investigation on the binding mode of celastrol and CHEK1 protein (ID: 4FSN). **(A)** predicted the binding site between celastrol and CHEK1; **(B)** showed the binding pocket between celastrol and CHEK1, where the carboxyl group was exposed to the solvent environment; **(C)** predicted the interactions between celastrol and CHEK1 protein.

The above research confirmed that PROTAC YX-112 of celastrol could effectively degrade CHEK1 protein. Therefore, we predicted the binding mode between celastrol and CHEK1 using Discovery Studio v3.5 software to explore their binding information. Based on the docking results, we found that celastrol could bind to the active site of CHEK1 protein (Figure 7A). Analysis of the binding pocket revealed that the carboxyl group on celastrol was exposed to the solvent environment, providing a suitable binding site for PROTAC molecule (Figure 7B. Interaction analysis revealed that celastrol could form hydrogen bond interaction with key residue GLN13, and form van der Waals forces interactions with residues THR13, GLY16, ALA36, TYR86, CYS87, GLY90, GLU91, ASP94, GLU134, ASN135, SER147 and ASP148 (Figure 7C). Therefore, celastrol and CHEK1 had a certain binding ability. Moreover, these docking results could provide favorable support for the effective degradation of CHEK1 protein by PROTAC YX-112 of celastrol.

## 4 Conclusion

Triple-negative breast cancer is the most aggressive, most easily metastasized and worst prognosis malignant tumor in breast cancer. Therefore, it is an urgent research work to find a new treatment strategy for triple-negative breast cancer. Celastrol is the active ingredient of the plant Tripterygium wilfordii Hook. f., which has therapeutic effects on a variety of cancers, including triple-negative breast cancer. However, the therapeutic window of celastrol is narrow, and its mechanism of action in triple-negative breast cancer is not very clear. In recent years, PROTAC technology has been widely used in drug development, highlighting its advantages in enhancing activity and reducing toxicity. Combined with proteomics technology, it can effectively identify the targets of natural products. In this study, we developed a novel PROTAC YX-112 of celastrol using the PROTAC strategy, which had an IC_50_ value of 0.32 ± 0.04 μM against triple-negative breast cancer MDA-MB-231 cells, and its antiproliferative activity was 3 times that of celastrol, demonstrating high potential for application. Subsequently, using DIA-based proteomics technology, the molecular mechanism of YX-112 in MDA-MB-231 cells was revealed through differential protein, GO enrichment, KEGG enrichment, and PPI analyses. Subsequently, Western blot experiments confirmed that YX-112 could target the degradation of CHEK1 and PIK3R2 proteins in MDA-MB-231 cells in a ubiquitin-proteasome dependent manner. YX-112 could significantly downregulate the expression levels of CDK4 and p-AKT, and the downregulation intensity was stronger than that of celastrol, indicating that YX-112 could exert its effects by regulating the cell cycle and PI3K-AKT-mTOR pathway. Finally, molecular docking predicted the binding mode between celastrol and CHEK1 protein, showing that celastrol could form hydrogen bond interaction with the key residue GLN13. In summary, our study provides new strategies for exploring the derivation of natural products and their mechanisms of action.

## Data Availability

The original contributions presented in the study are included in the article/Supplementary Material, further inquiries can be directed to the corresponding authors.
