# Supplementary material for: YX-112, a novel celastrol-derived PROTAC, inhibits the development of triple-negative breast cancer by targeting the degradation of multiple proteins
Source: Front Pharmacol. 2025 Apr 15;16:1571135. doi: 10.3389/fphar.2025.1571135 (PMC12037497; doi:10.3389/fphar.2025.1571135)
Supplement: Supplementary file 1 [file DataSheet1.doc]

**YX-112, a novel celastrol-derived PROTAC, inhibits the development of triple-negative breast cancer by targeting the degradation of multiple proteins**

Yongxue Gu^a^, Mengmeng Yang^a^, Wenbin Wang^a^, Lihua Li^b^, Ying Ma^c^, Wenshan Liu^b, d, *^, Qiang Zhao^e, *^

^a^Thyroid and Breast Medical Center, Weifang People's Hospital, Shandong Second Medical University, Weifang 261041, Shandong Province, China

^b^Clinical Research Center, Affiliated Hospital of Shandong Second Medical University, Weifang 261041, Shandong Province, China

^c^School of Pharmacy, Tianjin Medical University, Tianjin 300070, China.

^d^Shandong Key Laboratory of Medicine and Health (Clinical Applied Pharmacology), Department of Pharmacy, Affiliated Hospital of Shandong Second Medical University, Weifang 261041, Shandong Province, China

^e^Department of Anesthesiology, Weifang People's Hospital, Shandong Second Medical University, Weifang 261041, Shandong Province, China

^*^Correspondence to:

Wenshan Liu Email: [liuwenshan@sdsmu.edu.cn](mailto:liuwenshan@sdsmu.edu.cn)

Qiang Zhao Email: [zq13563651520@163.com](mailto:zq13563651520@163.com)

Table S1. Significantly downregulated and upregulated genes and their corresponding FC values and P Value were displayed.

| Number | Protein.Names | Genes | FC(YX-112 VS DMSO) | P Value |
| --- | --- | --- | --- | --- |
| 1 | P85B_HUMAN | PIK3R2 | 0.048627234 | 0.026297789 |
| 2 | CHK1_HUMAN | CHEK1 | 0 | 0.002716528 |
| 3 | AKA10_HUMAN | AKAP10 | 0.178783168 | 0.036716993 |
| 4 | SI1L3_HUMAN | SIPA1L3 | 0.200076981 | 0.039570783 |
| 5 | FZD6_HUMAN | FZD6 | 0.312490562 | 0.020818002 |
| 6 | OGA_HUMAN | OGA | 0.572944009 | 0.032441886 |
| 7 | WDR1_HUMAN | WDR1 | 0.668304027 | 0.048141688 |
| 8 | IPO13_HUMAN | IPO13 | 0.467272549 | 0.046234802 |
| 9 | CCNB2_HUMAN | CCNB2 | 0.297787335 | 0.037352649 |
| 10 | YIF1A_HUMAN | YIF1A | 0.393948971 | 0.043198284 |
| 11 | TACC2_HUMAN | TACC2 | 0.160255332 | 0.032314215 |
| 12 | ERCC1_HUMAN | ERCC1 | 0 | 0.00147995 |
| 13 | HMOX1_HUMAN | HMOX1 | > 10 | 0.004042231 |
| 14 | C1TC_HUMAN | MTHFD1 | 0.630268796 | 0.035180328 |
| 15 | CDK4_HUMAN | CDK4 | 0.619400787 | 0.014260956 |
| 16 | ATF2_HUMAN | ATF2 | 4.447685383 | 0.036693331 |
| 17 | PHKG2_HUMAN | PHKG2 | 0 | 4.36736E-06 |
| 18 | ARY1_HUMAN | NAT1 | 0.191995863 | 0.033124691 |
| 19 | DCK_HUMAN | DCK | 0.589045998 | 0.02184204 |
| 20 | RXRB_HUMAN | RXRB | 0 | 0.000761204 |
| 21 | CCND3_HUMAN | CCND3 | 0 | 0.00242571 |
| 22 | SC6A6_HUMAN | SLC6A6 | 0.510256407 | 0.048147993 |
| 23 | MCM7_HUMAN | MCM7 | 0.508703075 | 0.047505872 |
| 24 | COIA1_HUMAN | COL18A1 | 0 | 0.005312044 |
| 25 | VHL_HUMAN | VHL | 0 | 0.007973384 |
| 26 | ID1_HUMAN | ID1 | 0 | 0.001205636 |
| 27 | UBA7_HUMAN | UBA7 | 0.194154214 | 0.037361588 |
| 28 | CSK_HUMAN | CSK | 0.652863183 | 0.014099502 |
| 29 | FAS_HUMAN | FASN | 0.523982448 | 0.017092706 |
| 30 | CDK7_HUMAN | CDK7 | 0.612374422 | 0.03895411 |
| 31 | RAB9A_HUMAN | RAB9A | 0.156841651 | 0.011181802 |
| 32 | CDK5_HUMAN | CDK5 | 0.512352503 | 0.04578333 |
| 33 | NFKB2_HUMAN | NFKB2 | 0.590001372 | 0.031571133 |
| 34 | AMPD3_HUMAN | AMPD3 | 0 | 7.86356E-05 |
| 35 | TLE3_HUMAN | TLE3 | 0.620823492 | 0.048697918 |
| 36 | SPIR1_HUMAN | SPIRE1 | 0.364482077 | 0.045211357 |
| 37 | MZT1_HUMAN | MZT1 | 6.915313827 | 0.00756556 |
| 38 | WASC5_HUMAN | WASHC5 | 0.321069587 | 0.038340702 |
| 39 | CAF1B_HUMAN | CHAF1B | 0 | 0.007345078 |
| 40 | MYO9B_HUMAN | MYO9B | 0 | 0.008594875 |
| 41 | DYR1A_HUMAN | DYRK1A | 0 | 0.003925076 |
| 42 | AUHM_HUMAN | AUH | 0 | 0.010119687 |
| 43 | SEPT6_HUMAN | SEPTIN6 | 0 | 0.001154936 |
| 44 | KEAP1_HUMAN | KEAP1 | 0.507926528 | 0.035187552 |
| 45 | EFR3A_HUMAN | EFR3A | 0 | 4.82861E-06 |
| 46 | PDIA5_HUMAN | PDIA5 | 0.201599282 | 0.045328142 |
| 47 | UBP10_HUMAN | USP10 | 0.667018345 | 0.021819823 |
| 48 | PAF15_HUMAN | PCLAF | 1.568933586 | 0.005965266 |
| 49 | SETB1_HUMAN | SETDB1 | 0 | 0.004949035 |
| 50 | F263_HUMAN | PFKFB3 | 0.211933498 | 0.041529481 |
| 51 | ERC6L_HUMAN | ERCC6L | 0.65442398 | 0.012604146 |
| 52 | ATG2A_HUMAN | ATG2A | 0.668034427 | 0.041639251 |
| 53 | AP5B1_HUMAN | AP5B1 | 0.21436481 | 0.043974446 |
| 54 | PDZ11_HUMAN | PDZD11 | 1.715205671 | 0.024320614 |
| 55 | HECD3_HUMAN | HECTD3 | 0.535915676 | 0.022786282 |
| 56 | FHI2A_HUMAN | FHIP2A | 0.480046148 | 0.031461667 |
| 57 | TENS3_HUMAN | TNS3 | 0.477878307 | 0.013868615 |
| 58 | STRAA_HUMAN | STRADA | 0 | 0.00182424 |
| 59 | MON2_HUMAN | MON2 | 0.656812641 | 0.043740421 |
| 60 | ZER1_HUMAN | ZER1 | 0.22913884 | 0.045581525 |
| 61 | PATL1_HUMAN | PATL1 | 0.65138891 | 0.01998222 |
| 62 | DPP9_HUMAN | DPP9 | 0.62167023 | 0.04986664 |
| 63 | RPTOR_HUMAN | RPTOR | 0.262727302 | 0.049816252 |
| 64 | ARG28_HUMAN | ARHGEF28 | 0 | 0.01321561 |
| 65 | MTMRE_HUMAN | MTMR14 | 0.624620648 | 0.016177768 |
| 66 | PDPR_HUMAN | PDPR | 0.490400461 | 0.011260895 |
| 67 | PEPL1_HUMAN | NPEPL1 | 0.614045812 | 0.025803085 |
| 68 | SHCBP_HUMAN | SHCBP1 | 0 | 0.016498066 |
| 69 | CX038_HUMAN | CXorf38 | 0.58411187 | 0.023430287 |
| 70 | NEK9_HUMAN | NEK9 | 0.546397317 | 0.002700307 |
| 71 | PCNP_HUMAN | PCNP | 1.52732518 | 0.045328729 |
| 72 | GBF1_HUMAN | GBF1 | 0.584383135 | 0.042020344 |
| 73 | RGP1_HUMAN | RGP1 | 0 | 0.008406586 |
| 74 | PGTA_HUMAN | RABGGTA | 0.565027202 | 0.044702872 |
| 75 | PTPRU_HUMAN | PTPRU | 0 | 0.002172207 |
| 76 | DVL3_HUMAN | DVL3 | 0 | 0.002180842 |
| 77 | MOCOS_HUMAN | MOCOS | 0.227031504 | 0.047569775 |
| 78 | RNF31_HUMAN | RNF31 | 0 | 0.001820915 |
| 79 | INP4A_HUMAN | INPP4A | 0.520461963 | 0.033701674 |
| 80 | SIN3A_HUMAN | SIN3A | 0.560705509 | 0.048636788 |
| 81 | METH_HUMAN | MTR | 0.411173097 | 0.02529193 |
| 82 | PI51A_HUMAN | PIP5K1A | 0 | 0.006263882 |
| 83 | MIPEP_HUMAN | MIPEP | 0 | 5.84747E-07 |
| 84 | LST8_HUMAN | MLST8 | 0.22630152 | 0.038125541 |
| 85 | SYTM_HUMAN | TARS2 | 0 | 0.004485263 |
| 86 | RPAP1_HUMAN | RPAP1 | 0.333631689 | 0.028053877 |
| 87 | XPO4_HUMAN | XPO4 | 0.171922377 | 0.018290716 |
| 88 | BORG4_HUMAN | CDC42EP4 | 4.548515036 | 0.04848249 |
| 89 | IFG15_HUMAN | TOR1AIP2 | 0.467289518 | 0.033813914 |
| 90 | E41L1_HUMAN | EPB41L1 | 0.213903309 | 0.025373731 |
| 91 | SMRCD_HUMAN | SMARCAD1 | 0.147696369 | 0.01900126 |
| 92 | CAPAM_HUMAN | PCIF1 | 0.108043173 | 0.001295468 |
| 93 | DOCK5_HUMAN | DOCK5 | 0.616404274 | 0.043297103 |
| 94 | RPF1_HUMAN | RPF1 | 0 | 0.010833079 |
| 95 | RPA2_HUMAN | POLR1B | 0.200512993 | 0.037806424 |
| 96 | EPG5_HUMAN | EPG5 | 0 | 0.002323093 |
| 97 | KIF15_HUMAN | KIF15 | 0 | 0.006560121 |
| 98 | HOME3_HUMAN | HOMER3 | 0.248383228 | 0.040323857 |
| 99 | PANK4_HUMAN | PANK4 | 0.303295964 | 0.040184122 |
| 100 | UCKL1_HUMAN | UCKL1 | 0.536362845 | 0.015907969 |
| 101 | CWC25_HUMAN | CWC25 | > 10 | 0.000115627 |
| 102 | BABA2_HUMAN | BABAM2 | 0.636605835 | 0.046561514 |
| 103 | DTL_HUMAN | DTL | 0.241072745 | 0.043383002 |
| 104 | ORC3_HUMAN | ORC3 | 0 | 0.038059452 |
| 105 | SIX4_HUMAN | SIX4 | 0 | 0.020803385 |
| 106 | HACL1_HUMAN | HACL1 | 0 | 1.64753E-06 |
| 107 | PSF2_HUMAN | GINS2 | 1.506615697 | 0.041459886 |
| 108 | LRCH1_HUMAN | LRCH1 | 0 | 0.02062988 |
| 109 | SNX24_HUMAN | SNX24 | 0.650108445 | 0.020778151 |
| 110 | WASC3_HUMAN | WASHC3 | 2.243396604 | 0.014155726 |
| 111 | BOLA1_HUMAN | BOLA1 | 4.473643716 | 0.039120776 |
| 112 | PAXB1_HUMAN | PAXBP1 | 0.253430647 | 0.013307096 |


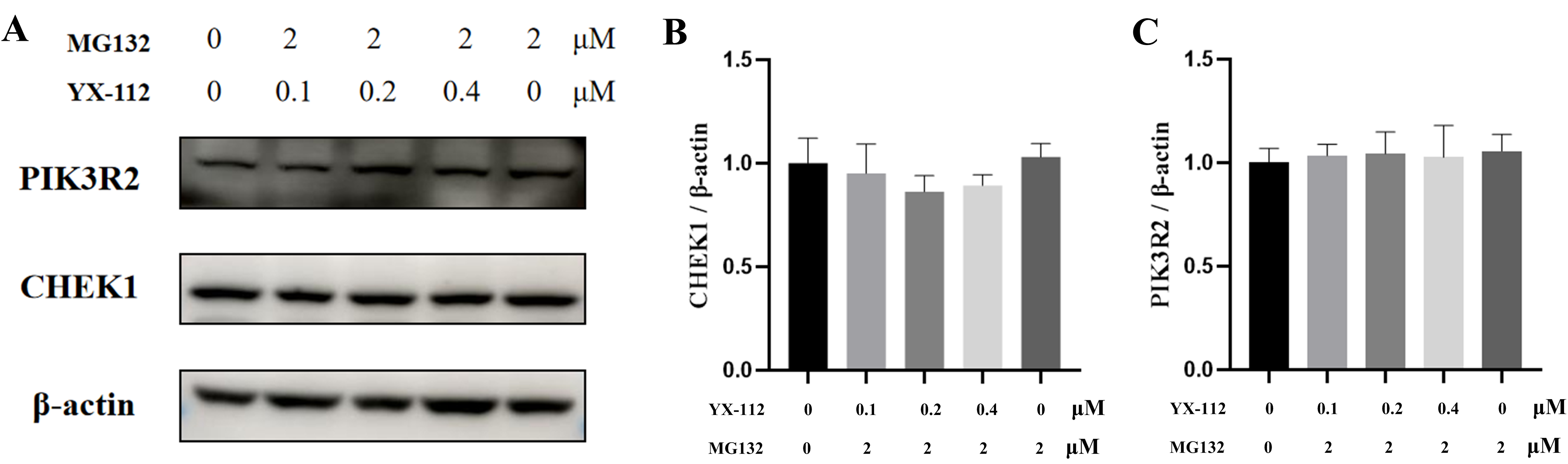


Figure S1. Validation of the degradation mechanism of compound YX-112. (A) Levels of CHEK1 and PIK3R2 in MDA-MB-231 cells treated with YX-112 (0, 0.1, 0.2, 0.4 μM) or co-treated with MG132 (2 μM) for 24 h. Data from three independent experiments; (B) Statistical analysis of three independent experiments.

**

**

Scheme S1. The synthetic route of compound YX-112. Reagents and conditions: (a) DIPEA, DMF, 90 ^o^C; (b) TFA, CH_2_Cl_2_, rt; (c) Compound 3, HATU, DIPEA, DMF, 60 ^o^C.

1. **The synthesis process of YX-112.**
2. (2,6-dioxopiperidin-3-yl)-5-fluoroisoindoline-1,3-dione (Compound 1, 2 mmol), *tert*-butyl 4-(piperazin-1-ylmethyl)piperidine-1-carboxylate (compound 2, 2.6 mmol), and DIPEA (6 mmol) were dissolved in DMF and reacted overnight at 90 ^o^C. After detecting the completion of the reaction through TLC, the solvent was removed under reduced pressure. The remaining mixture was extracted with CH_2_Cl_2_ (3 × 20 mL) and washed with saturated NaCl solution (3 × 20 mL). The merged organic phases were dried over anhydrous Na_2_SO_4_ and evaporated under reduced pressure to obtain the residues. Trifluoroacetic acid (12 mmol) and CH_2_Cl_2_ (15 mL) were added to the residues and stirred at 25 ^o^C for 0.5 h. After the completion of the reaction was detected by TLC, the solvent was removed under reduced pressure, and the pH was adjusted to alkaline by adding saturated sodium carbonate solution. The remaining mixture was extracted with CH_2_Cl_2_ (3 × 20 mL) and washed with saturated NaCl solution (3 × 20 mL). The merged organic phases were dried over anhydrous Na_2_SO_4_ and evaporated under reduced pressure to obtain the residues. The residues were purified by silica gel column chromatography (CH_2_Cl_2_: MeOH=20:1) to obtain intermediate 3.

Celastrol (1 mmol), Compound 3 (1.1 mmol), HATU (1.1 mmol), and DIPEA (3 mmol) were dissolved in DMF and reacted overnight at 60 ^o^C. After detecting the completion of the reaction through TLC, the solvent was removed under reduced pressure. The remaining mixture was extracted with CH_2_Cl_2_ (3 × 20 mL) and washed with saturated NaCl solution (3 × 20 mL). The merged organic phases were dried over anhydrous Na_2_SO_4_ and evaporated under reduced pressure to obtain the residues. The residues were purified by silica gel column chromatography (CH_2_Cl_2_: MeOH=20:1) to obtain product YX-112.

**1.1 2-(2,6-dioxopiperidin-3-yl)-5-(4-((1-((2R,4aS,6aS,12bR,14aS,14bR)-10-hydroxy-2,4a,6a,9,12b,14a-hexamethyl-11-oxo-1,2,3,4,4a,5,6,6a,11,12b,13,14,14a,14b-tetradecahydropicene-2-carbonyl)piperidin-4-yl)methyl)piperazin-1-yl)isoindoline-1,3-dione (YX-112)**

Yield: 21 %. Red solid, mp 176-178 ^o^C. ^1^H NMR (400 MHz, DMSO-*d*_6_) δ 11.10 (s, 1H), 8.77 (s, 1H), 7.68 (dd, *J* = 7.8 Hz, 1H), 7.34 (s, 1H), 7.26 (dd, *J* = 7.8 Hz, 1H), 7.10 (s, 1H), 6.48-6.30 (2H), 5.09 (d, *J* = 8.0 Hz, 1H), 4.45-4.20 (m, 2H), 3.60-3.45 (m, 10H), 2.95-2.82 (m, 1H), 2.65-2.57 (m, 1H), 2.22-2.08 (m, 5H), 2.06-1.96 (m, 2H), 1.87-1.68 (m, 6H), 1.60-1.50 (m, 2H), 1.45-1.33 (m, 5H), 1.32-1.15 (m, 13H), 1.10 (s, 3H), 0.85 (s, 3H), 0.47 (s, 3H). HRMS (ESI) calcd for C_48_H_59_N_3_O_8_ [M + H]^+^ 872.49622, found 872.49361.

1. **^1^H NMR spectra and HRMS of compound YX-112**


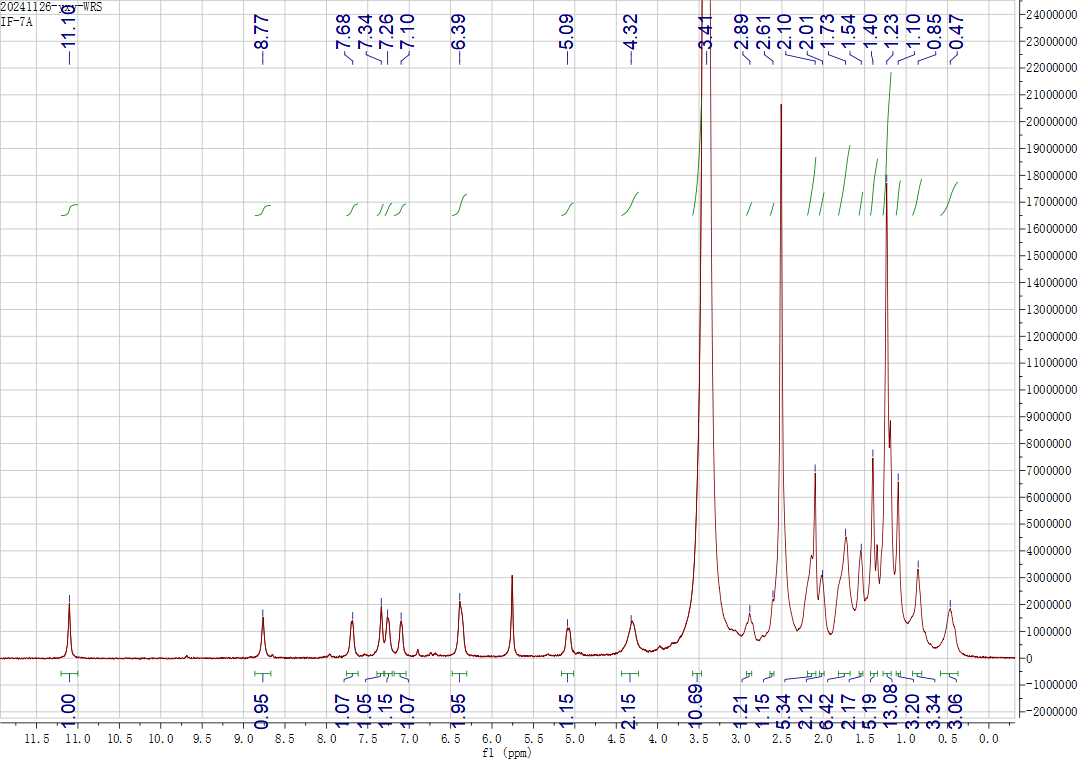


^1^H NMR spectrum of Compound 11h (DMSO-*d*_6_, 298 K, 400 MHz).

HRMS of Compound YX-112.
