# Supplementary figures and images for: YX-112, a novel celastrol-derived PROTAC, inhibits the development of triple-negative breast cancer by targeting the degradation of multiple proteins
Source: Front Pharmacol. 2025 Apr 15;16:1571135. doi: 10.3389/fphar.2025.1571135 (PMC12037497; doi:10.3389/fphar.2025.1571135)

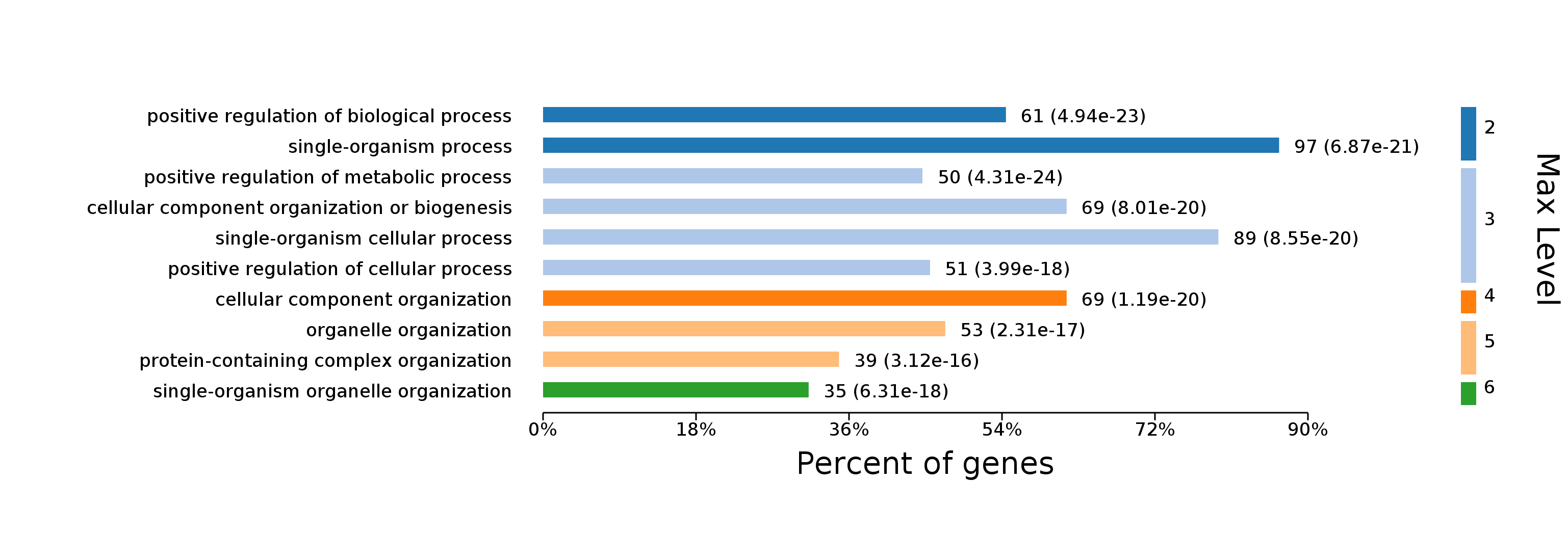

Supplement: Supplementary file 2 [file DataSheet2.zip › raw data/DIA Proteomics/GO/BP/bp_levels_bar.png]

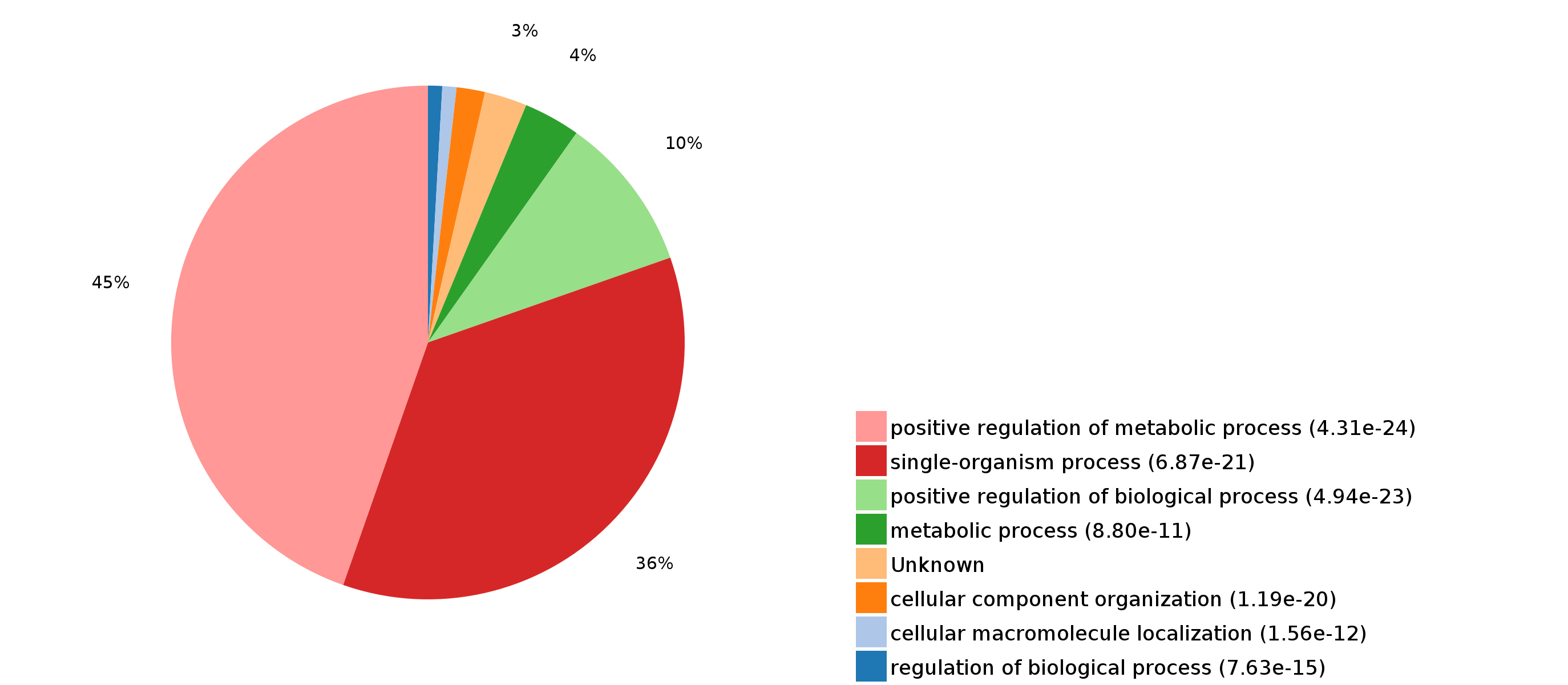

Supplement: Supplementary file 2 [file DataSheet2.zip › raw data/DIA Proteomics/GO/BP/bp_pie.png]

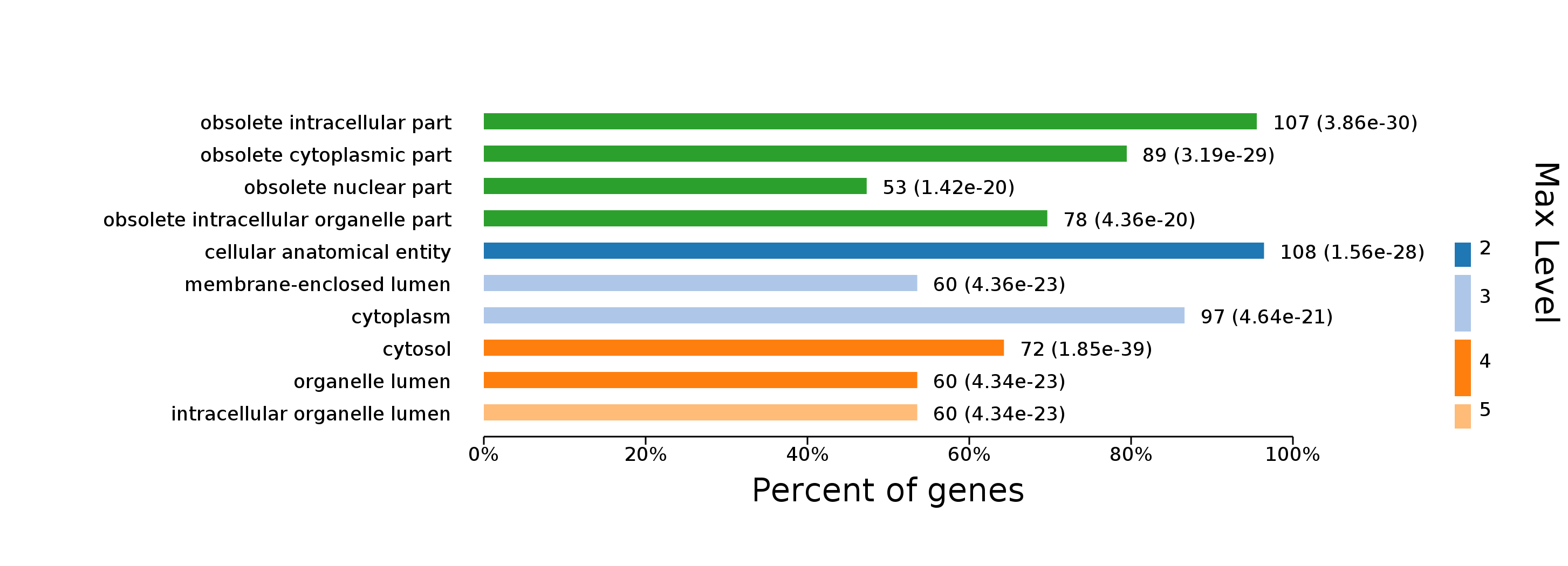

Supplement: Supplementary file 2 [file DataSheet2.zip › raw data/DIA Proteomics/GO/CC/cc_levels_bar.png]

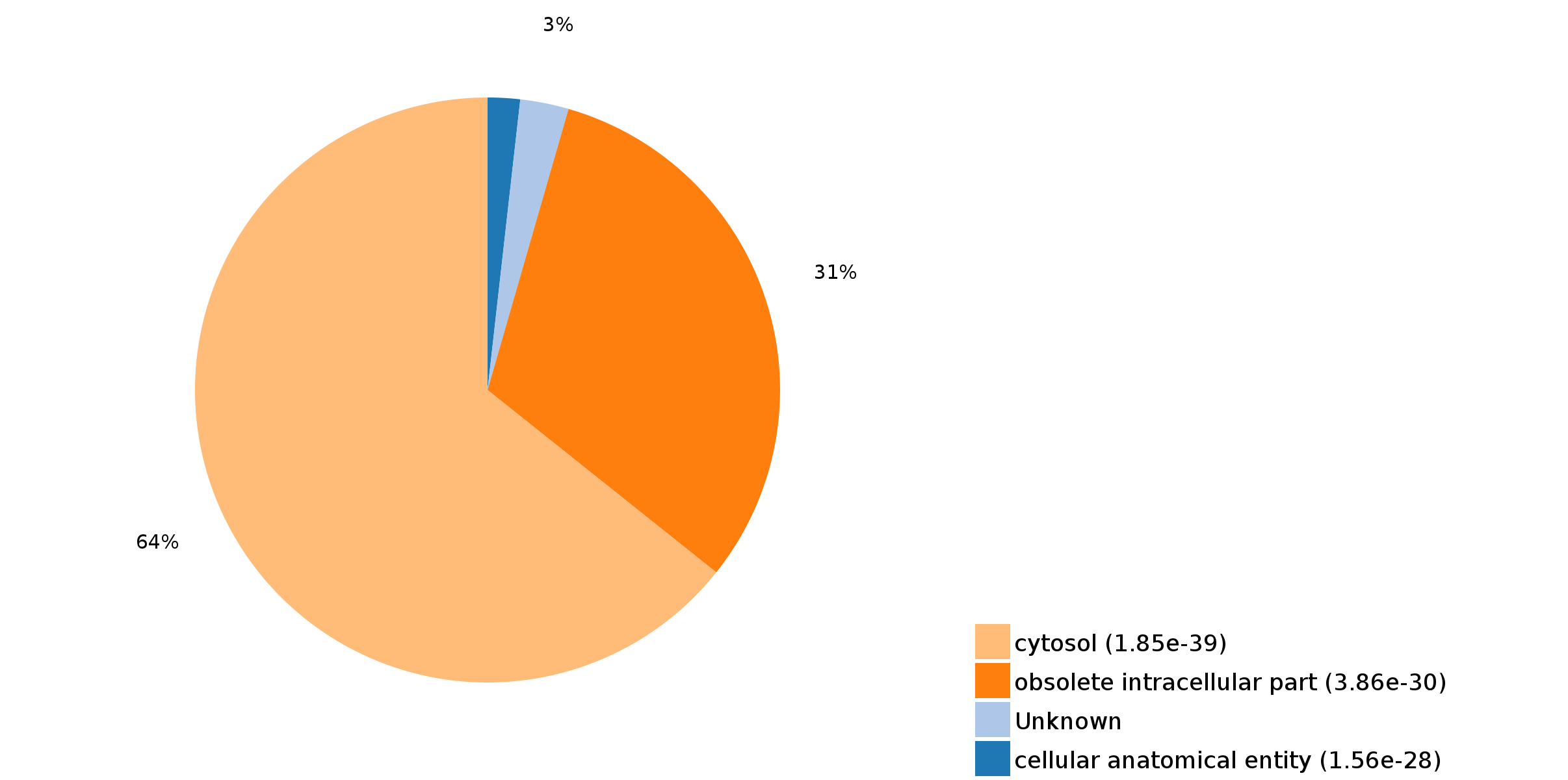

Supplement: Supplementary file 2 [file DataSheet2.zip › raw data/DIA Proteomics/GO/CC/cc_pie.png]

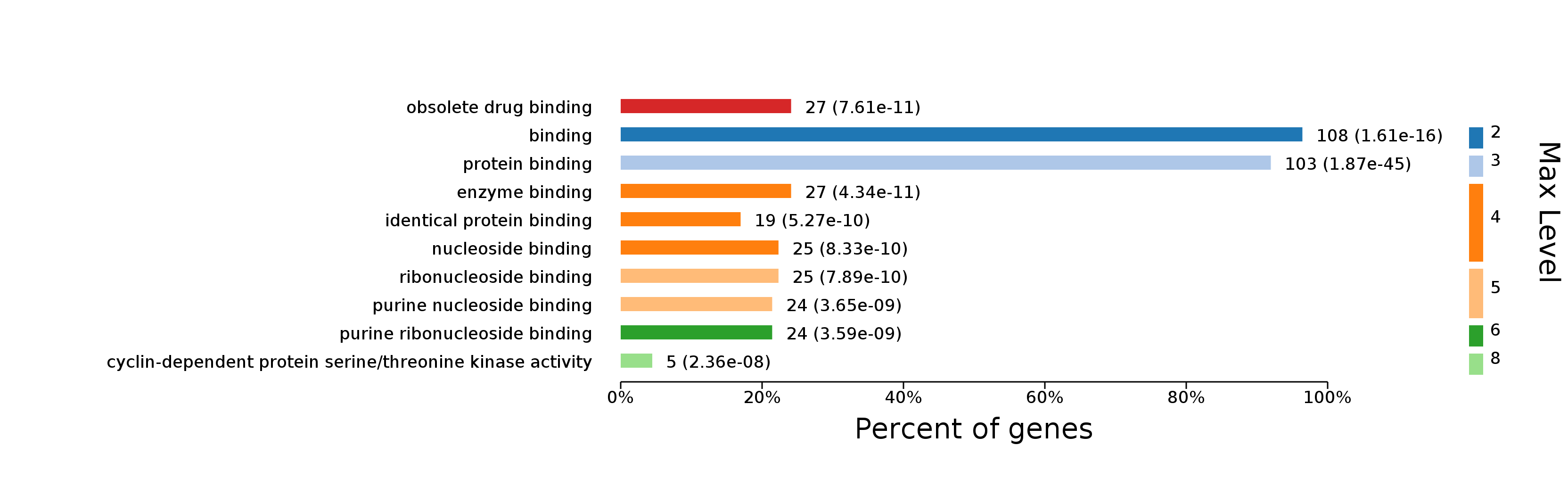

Supplement: Supplementary file 2 [file DataSheet2.zip › raw data/DIA Proteomics/GO/MF/mf_levels_bar.png]

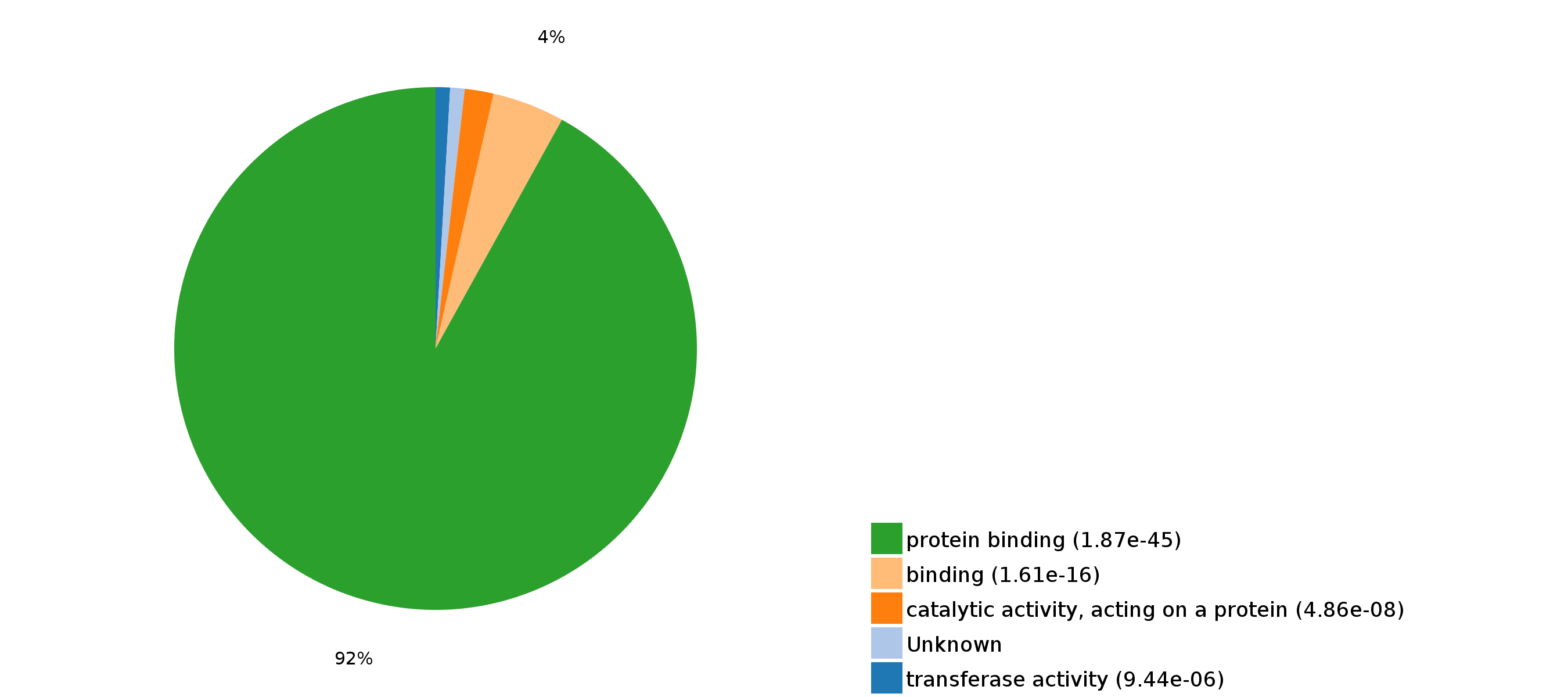

Supplement: Supplementary file 2 [file DataSheet2.zip › raw data/DIA Proteomics/GO/MF/mf_pie.png]

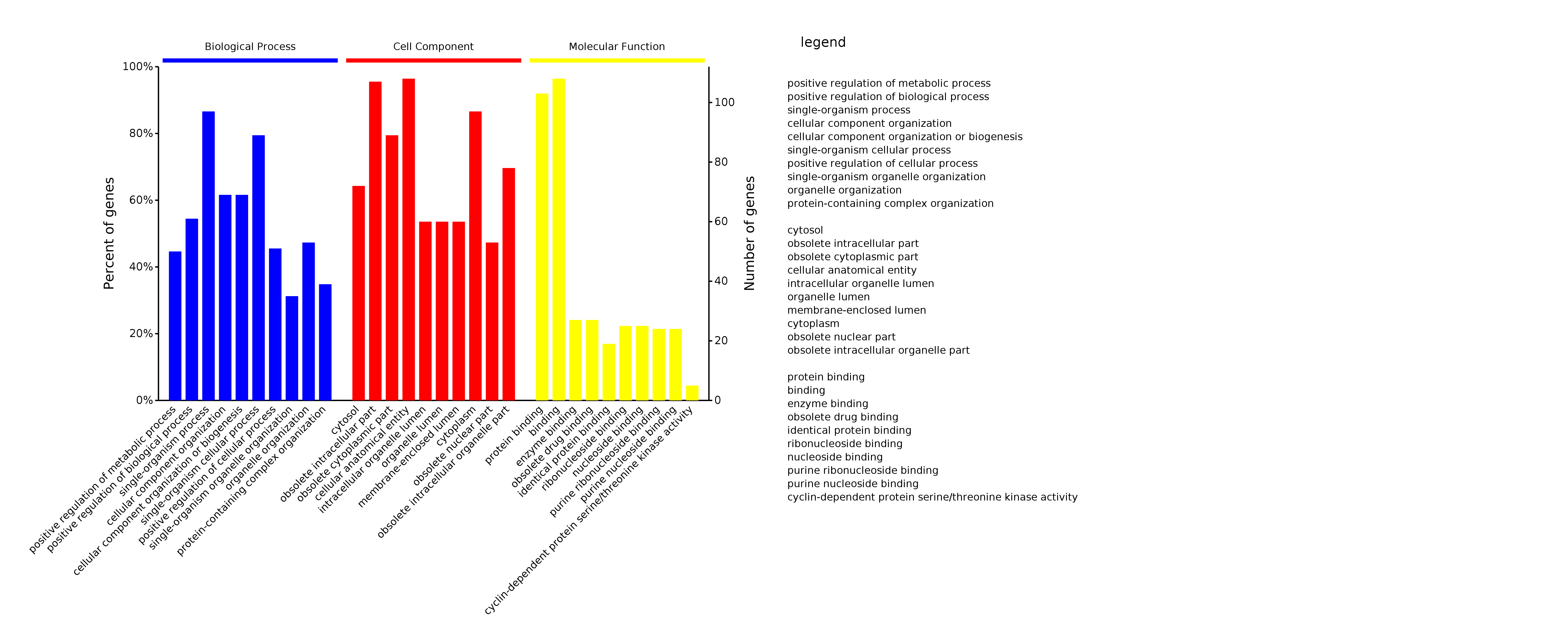

Supplement: Supplementary file 2 [file DataSheet2.zip › raw data/DIA Proteomics/GO/all_go_bar.png]

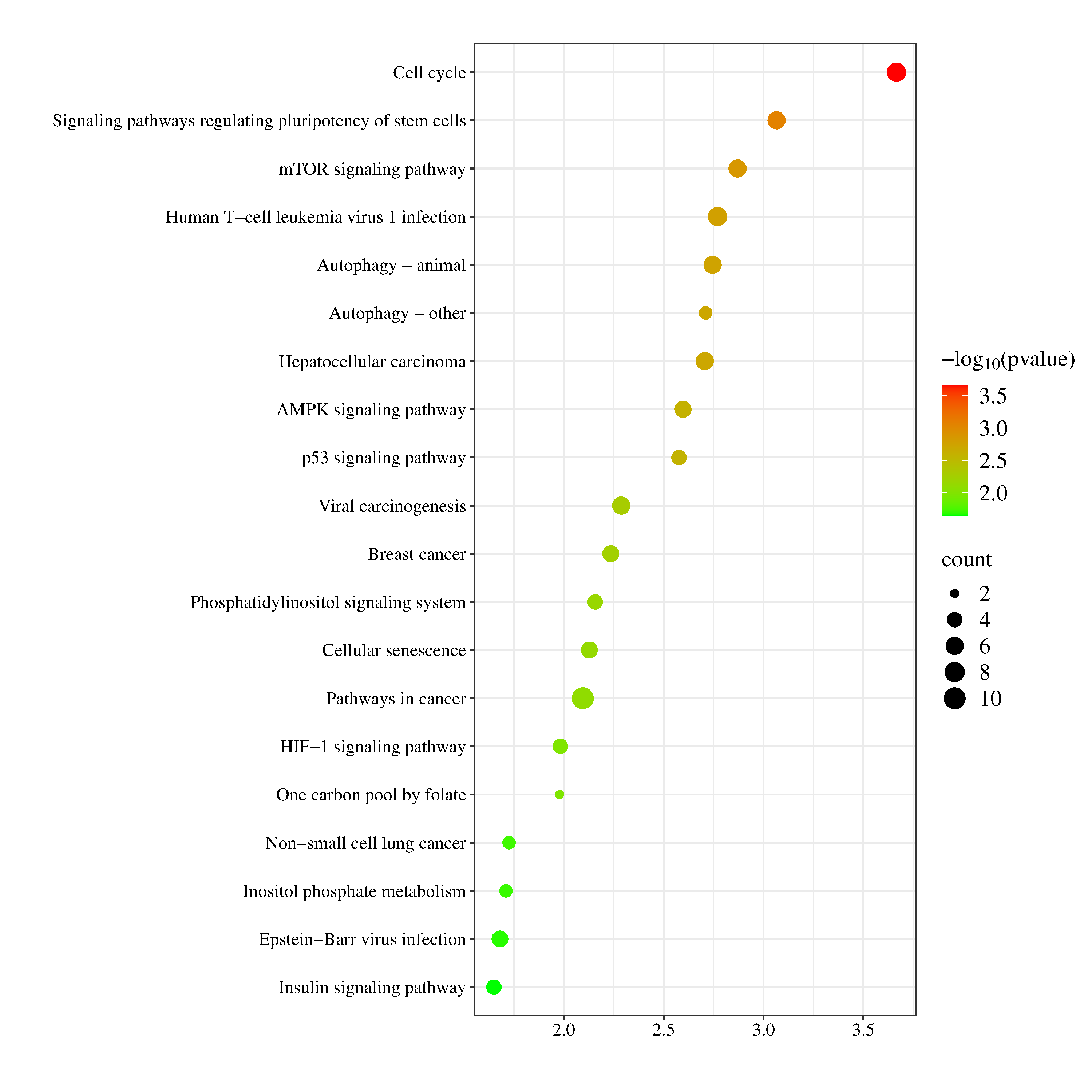

Supplement: Supplementary file 2 [file DataSheet2.zip › raw data/DIA Proteomics/KEGG/KEEG.png]

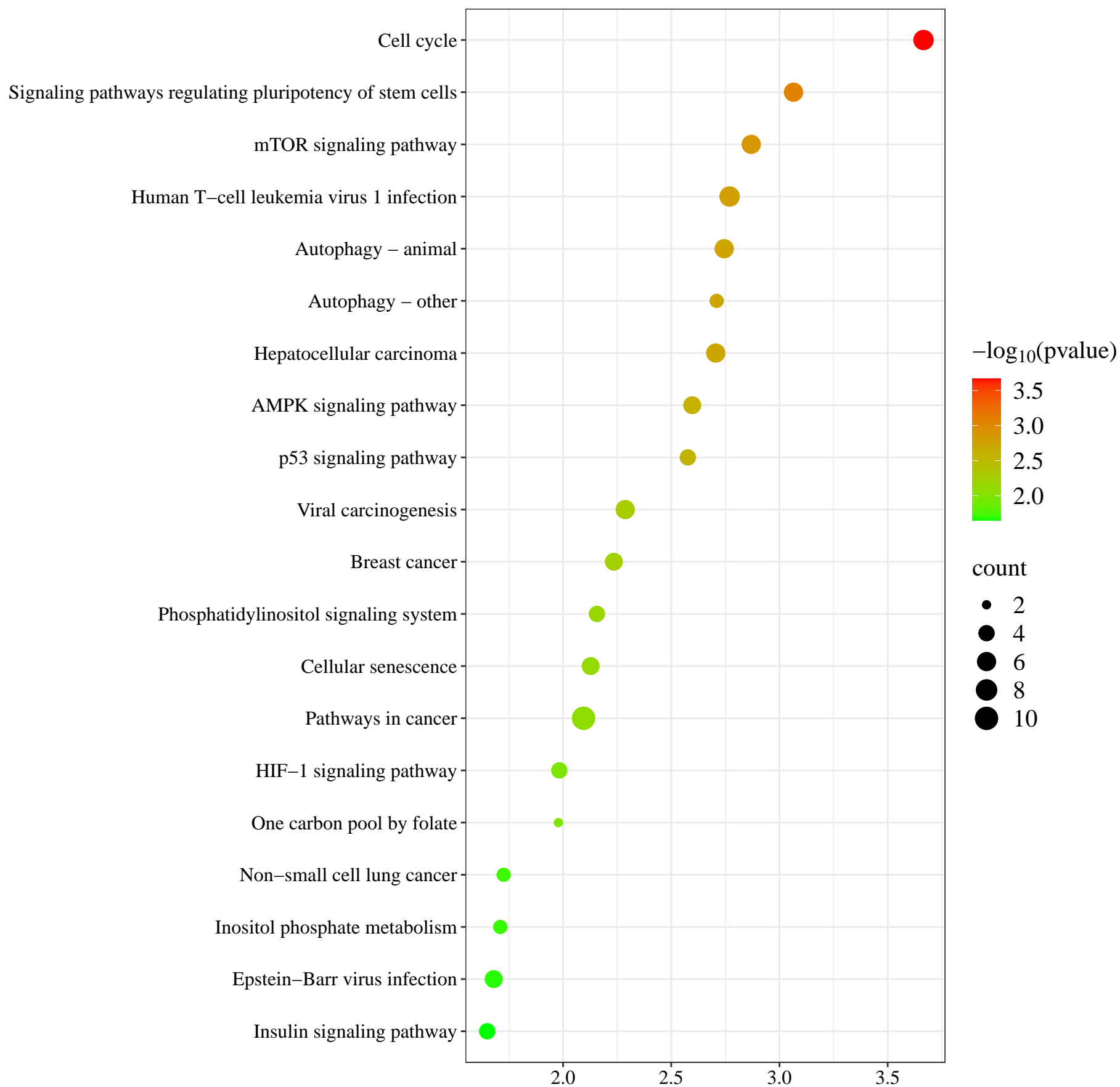

Supplement: Supplementary file 2 [file DataSheet2.zip › raw data/DIA Proteomics/KEGG/KEGG.pdf]

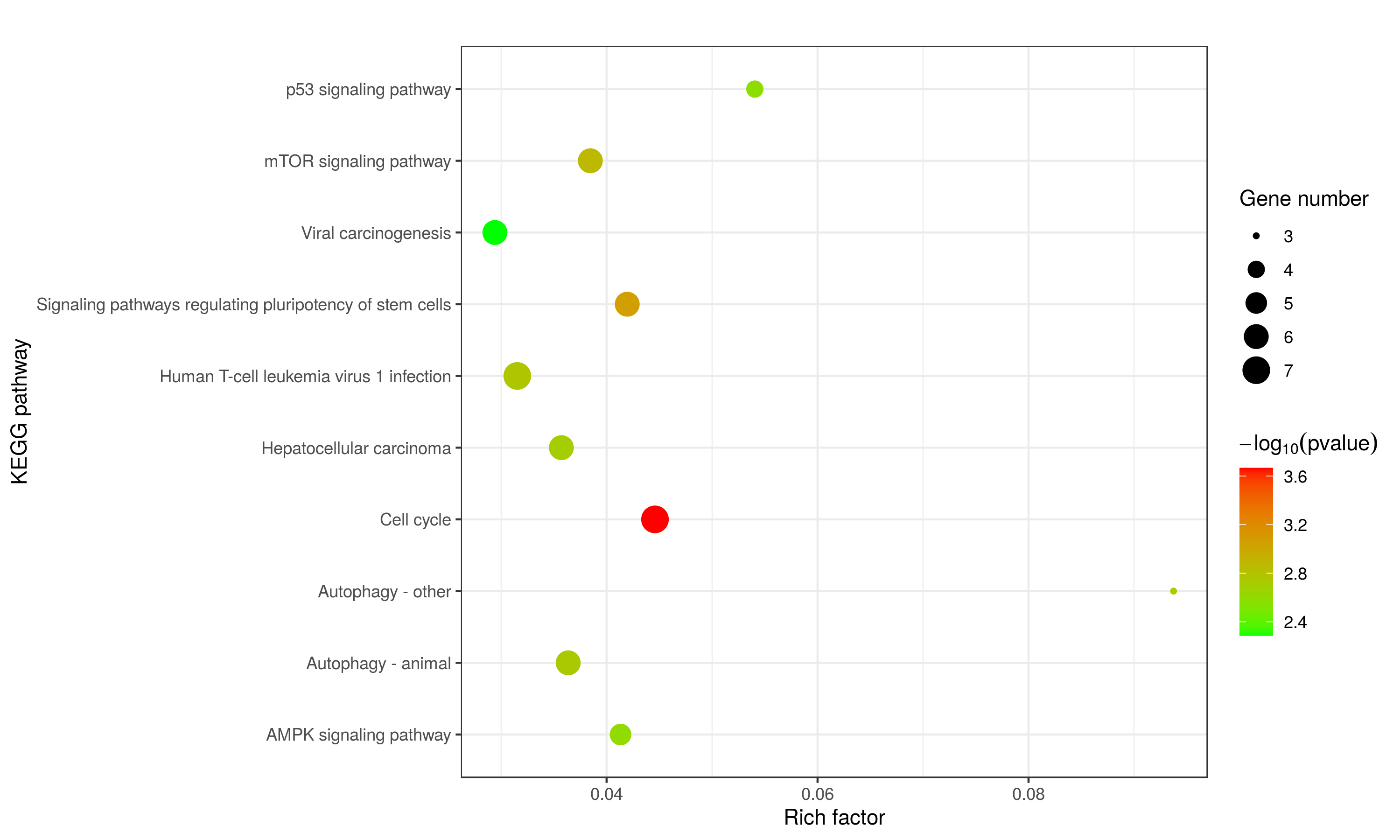

Supplement: Supplementary file 2 [file DataSheet2.zip › raw data/DIA Proteomics/KEGG/kegg240730231625.bubble.png]

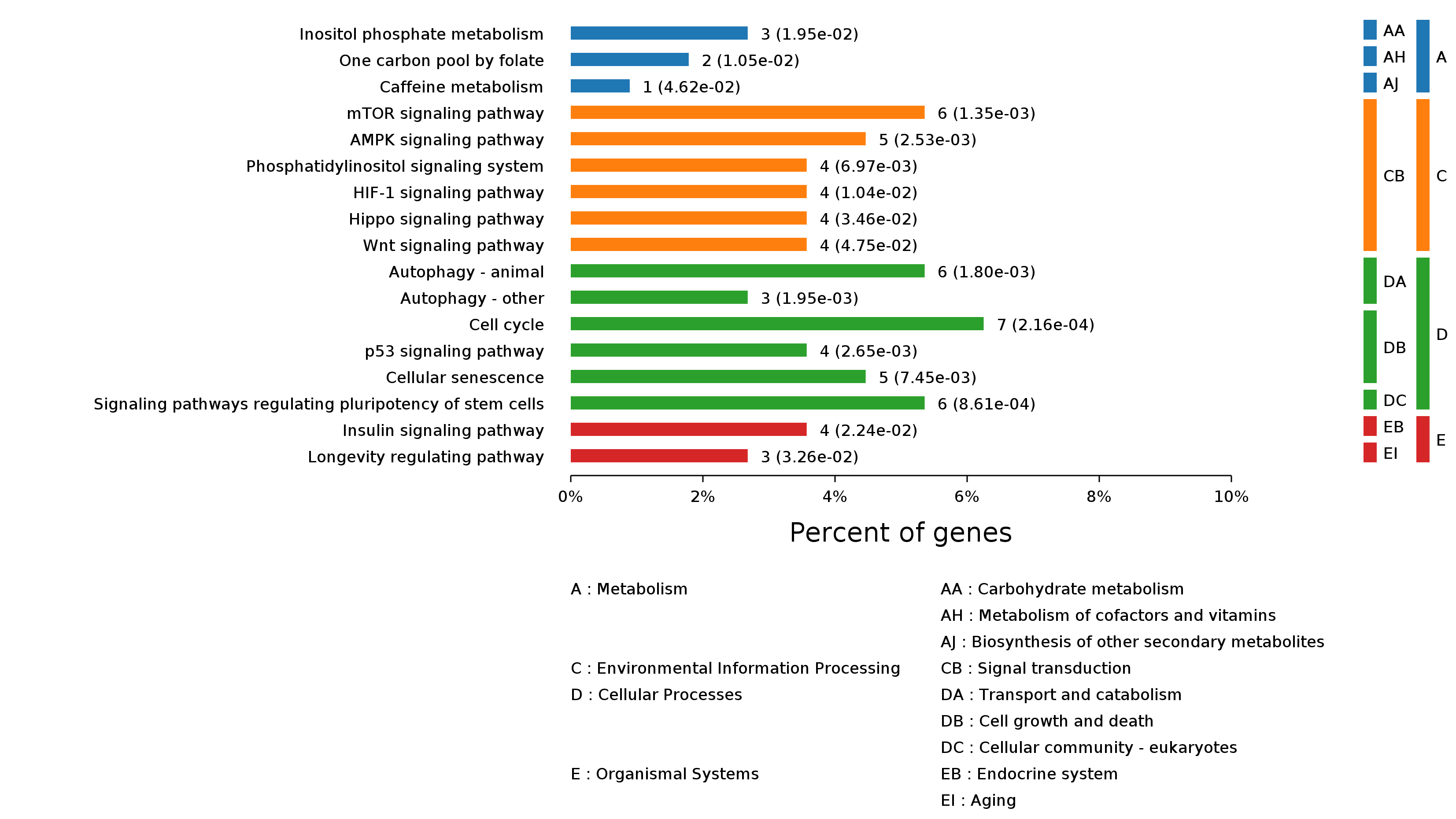

Supplement: Supplementary file 2 [file DataSheet2.zip › raw data/DIA Proteomics/KEGG/kegg_class.png]

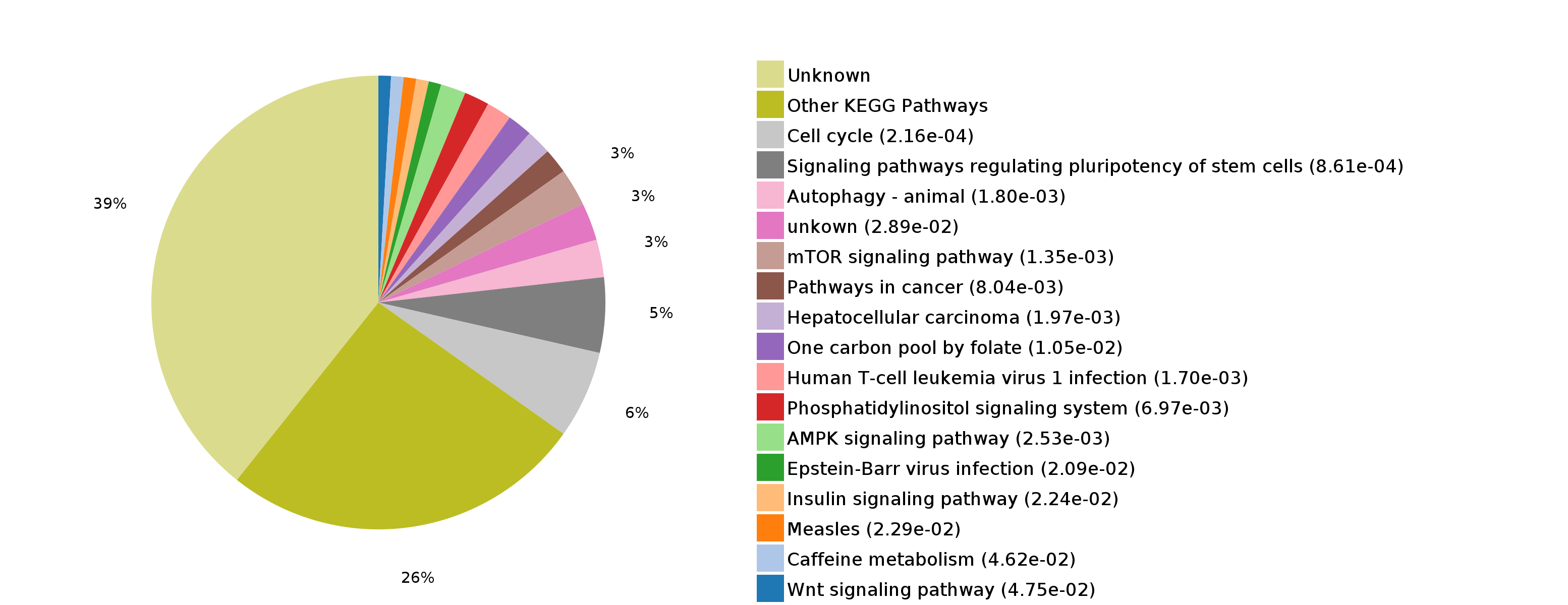

Supplement: Supplementary file 2 [file DataSheet2.zip › raw data/DIA Proteomics/KEGG/kegg_pie.png]

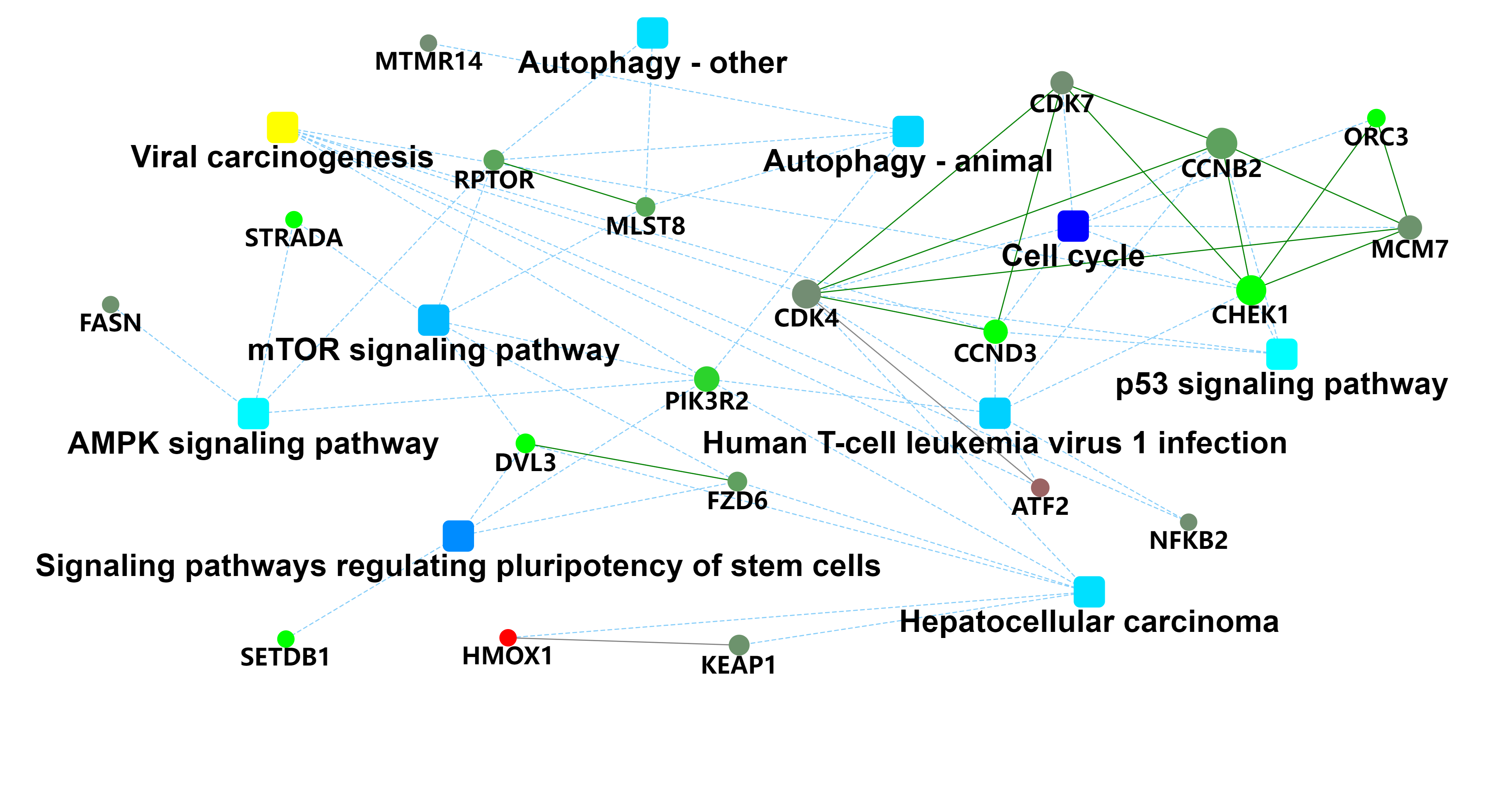

Supplement: Supplementary file 2 [file DataSheet2.zip › raw data/DIA Proteomics/PPI/ppi.png]

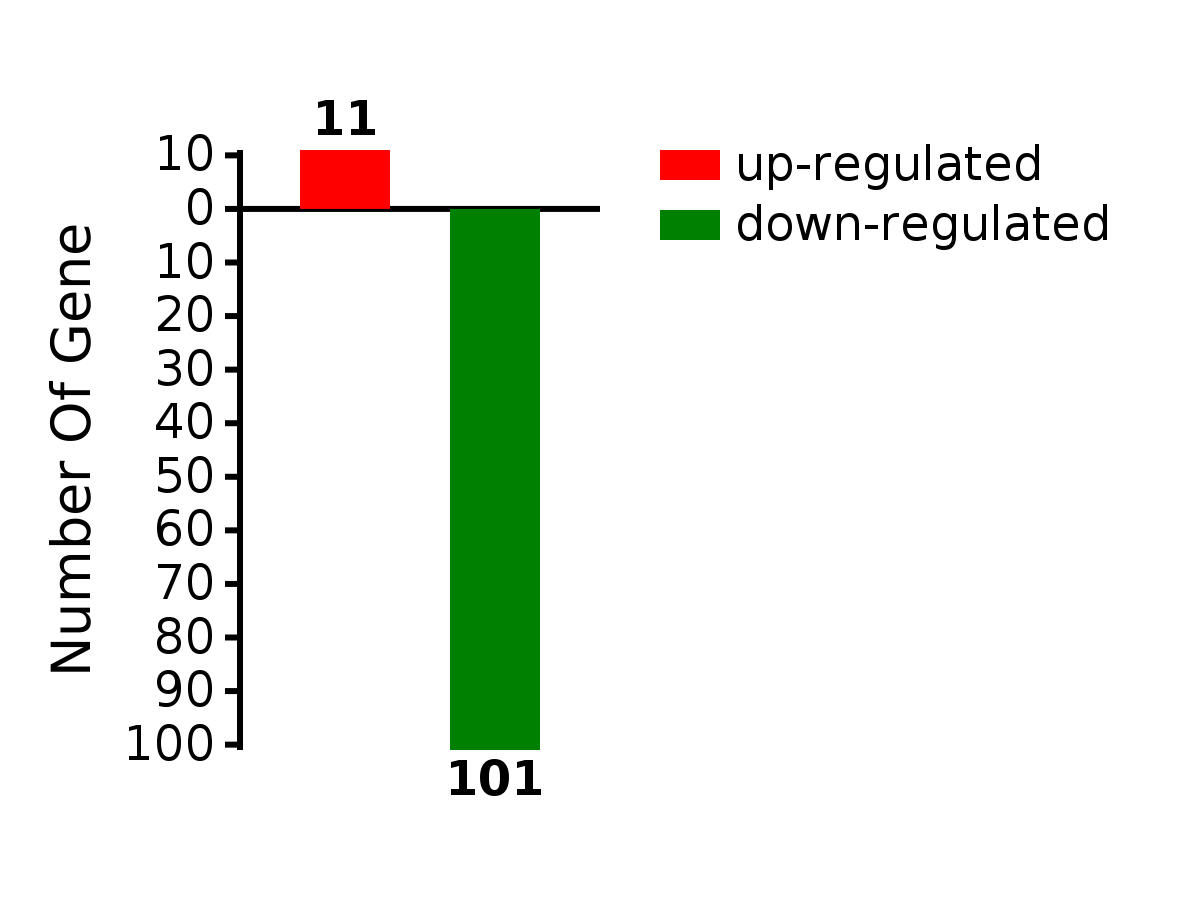

Supplement: Supplementary file 2 [file DataSheet2.zip › raw data/DIA Proteomics/foldchange_bar.png]

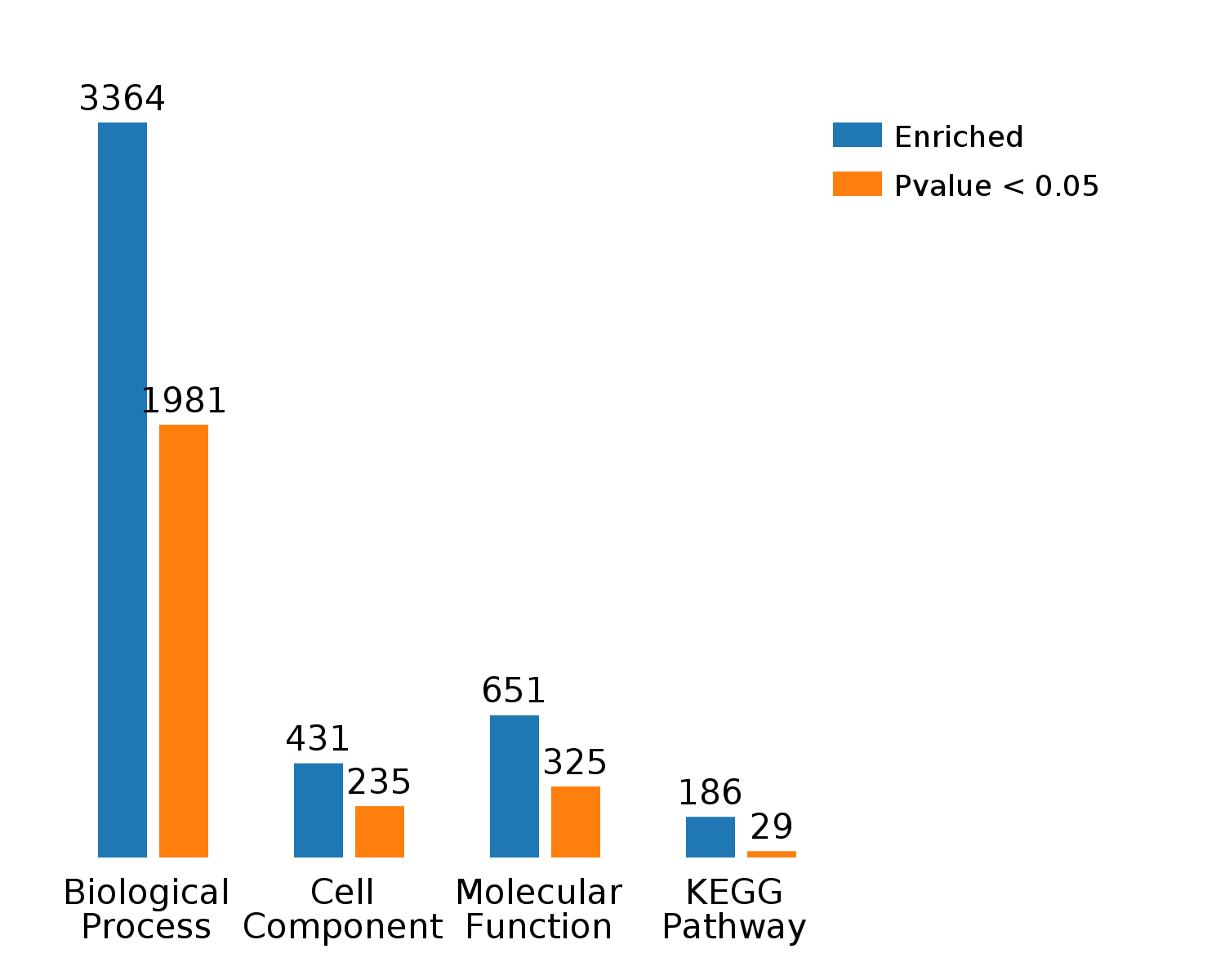

Supplement: Supplementary file 2 [file DataSheet2.zip › raw data/DIA Proteomics/statistical_summary.png]

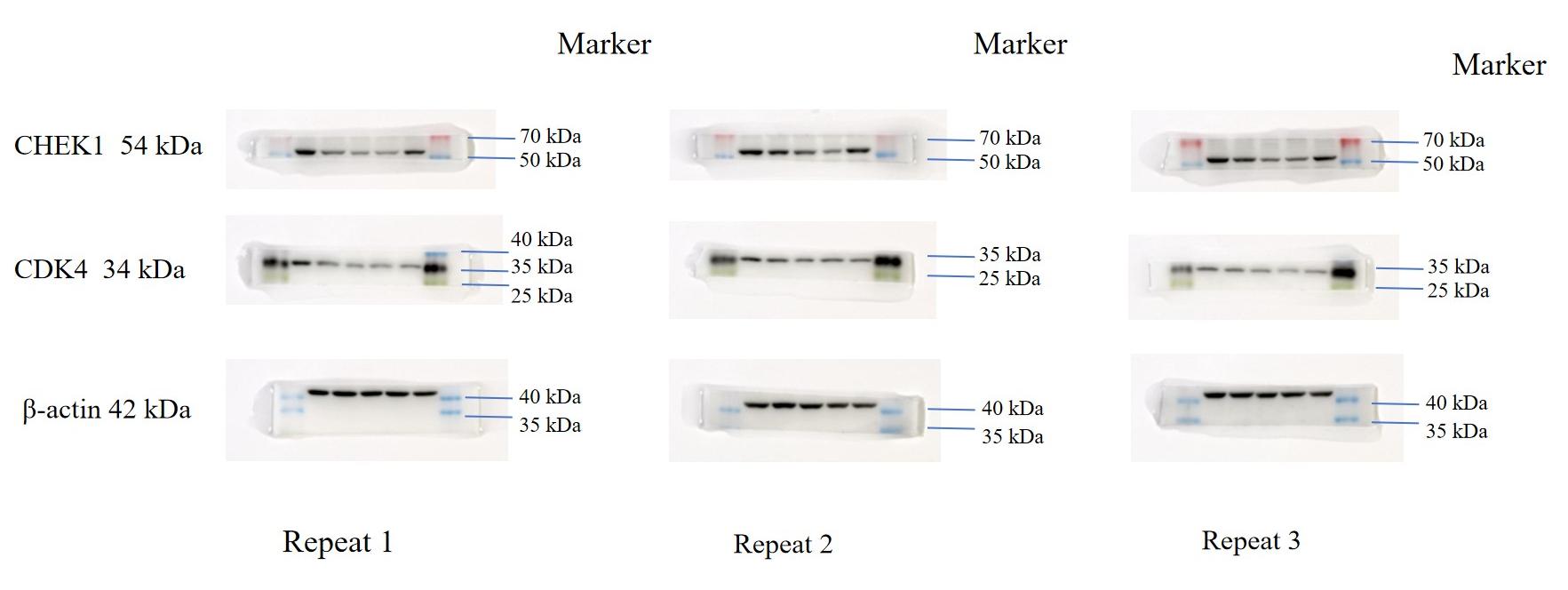

Supplement: Supplementary file 2 [file DataSheet2.zip › raw data/WB raw data/Raw data of Western Blots Images-Figure 6A.jpg]

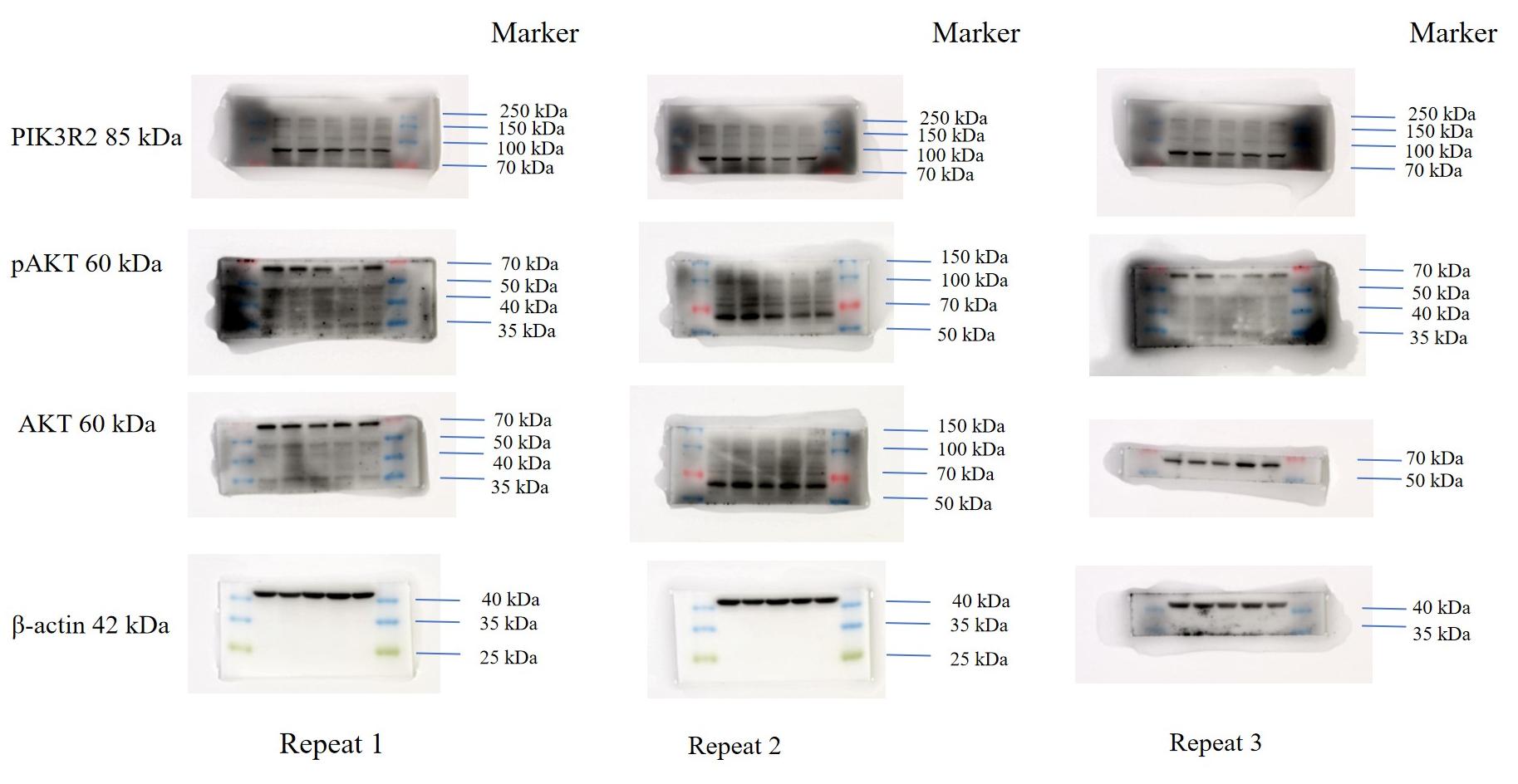

Supplement: Supplementary file 2 [file DataSheet2.zip › raw data/WB raw data/Raw data of Western Blots Images-Figure 6B.jpg]

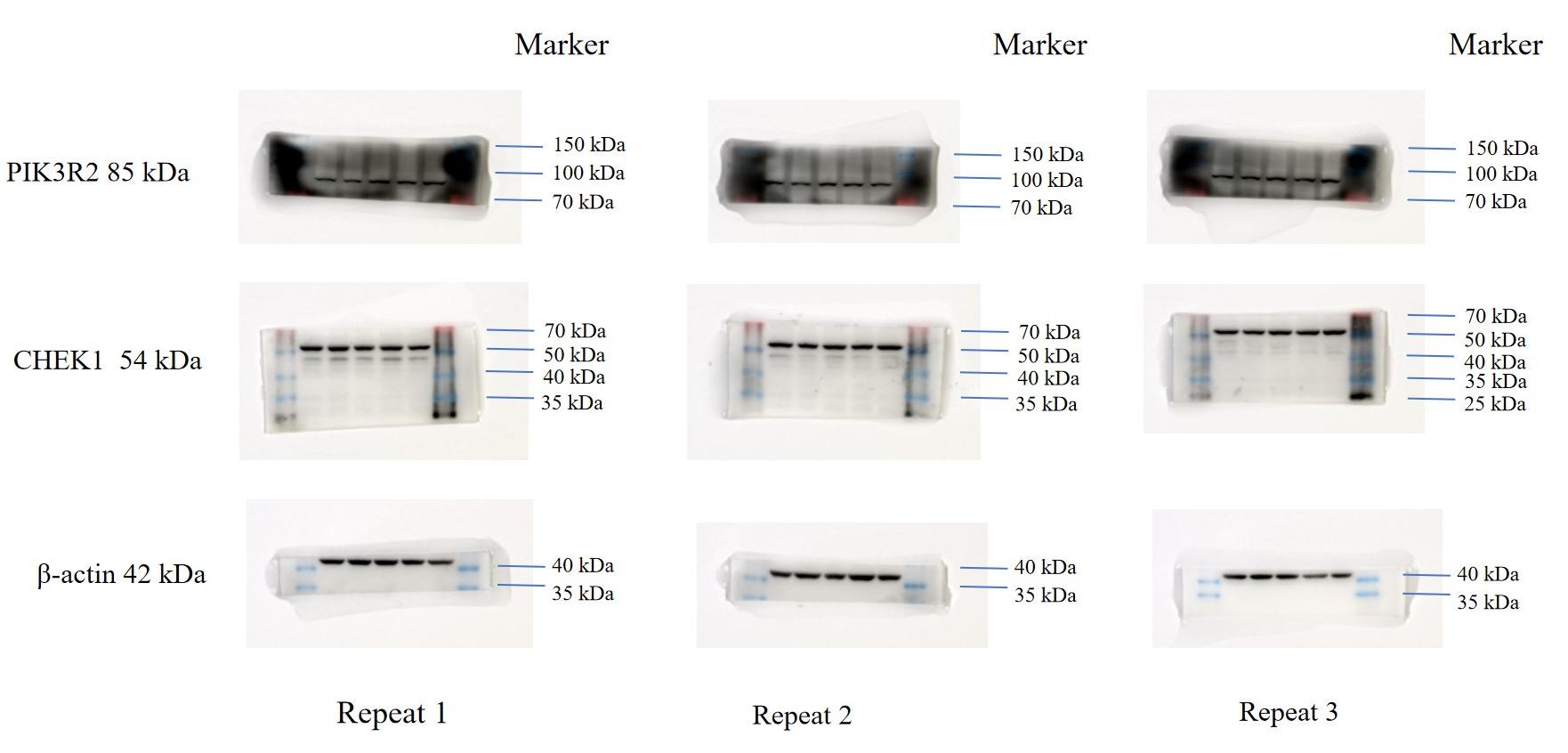

Supplement: Supplementary file 2 [file DataSheet2.zip › raw data/WB raw data/Raw data of Western Blots Images-Figure S1.jpg]
